# Modular Approach to Selected Configuration Interaction in an Arbitrary Spin Basis: Implementation and Comparison of Approaches

**DOI:** 10.1021/acs.jctc.3c00897

**Published:** 2023-12-07

**Authors:** Andrew W. Prentice, Jeremy P. Coe, Martin J. Paterson

**Affiliations:** Institute of Chemical Sciences, School of Engineering and Physical Sciences, Heriot-Watt University, Edinburgh EH14 4AS, U.K.

## Abstract

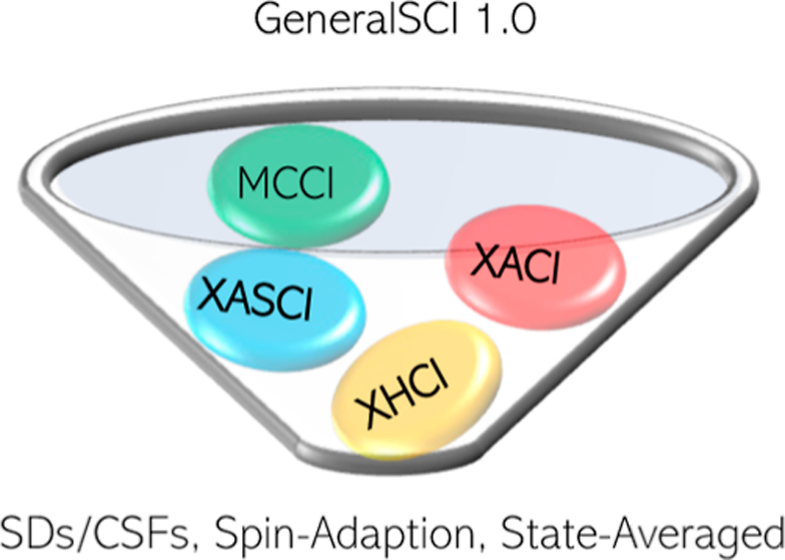

A modular selected configuration interaction (SCI) code has been developed that is based on the existing Monte-Carlo configuration interaction code (MCCI). The modularity allows various selection protocols to be implemented with ease and allows for fair comparison between wave functions built via different criteria. We have initially implemented adaptations of existing SCI theories, which are based on either energy- or coefficient-driven selection schemes. These codes have been implemented not only in the basis of Slater determinants (SDs) but also in the basis of configuration state functions (CSFs) and extended to state-averaged regimes. This allows one to take advantage of the reduced dimensionality of the wave function in the CSF basis and also the guarantee of pure spin states. All SCI methods were found to be able to predict potential energy surfaces to high accuracy, producing compact wave functions, when compared to full configuration interaction (FCI) for a variety of bond-breaking potential energy surfaces. The compactness of the error-controlled adaptive configuration interaction approach, particularly in the CSF basis, was apparent with nonparallelity errors within chemical accuracy while containing as little as 0.02% of the FCI CSF space. The size-to-accuracy was also extended to FCI spaces approaching one billion configurations.

## 1 Introduction

Accurately capturing the intricate nature of electron–electron interactions in atoms and molecules typically requires building upon the Hartree–Fock (HF) wave function to account for static and dynamic correlation. Ideally, one would subject a system of interest to a full configuration interaction (FCI) treatment, however, the factorial scaling, in terms of both the number of electrons (*n*) and basis functions (ϕ), of such an algorithm severely limits its application to all but the smallest of systems with widespread application to systems of arbitrary size essentially impossible for classical algorithms. Generally, FCI is used for small-molecule benchmarking,^1−20^ although various applications in few-electron systems have been reported.^21,22^

Selected configuration interaction (SCI) theories, stemming from the pioneering work of Huron, Malrieu, and Rancurel on the configuration interaction perturbatively selected iteratively (CIPSI) method,^23^ have been shown to accurately capture the electronic structure of complicated species while utilizing only a small portion of the FCI space, either in the Slater determinant (SD) or configuration state function (CSF) *n*-electron basis, thus widening the potential scope of FCI-quality calculations. The comparative blind-test study conducted by Eriksen and co-workers^24^ on the ground-state energy of C_6_H_6_, invoking the frozen-core approximation and using the cc-pVDZ basis set (*n* = 30, ϕ = 102), is a striking example of this. SCI theories have also been used to generate accurate potential energy surfaces (PES) for various bond breaking pathways.^25−38^ These compact unbiased wave functions, therefore, have promise for future work on dynamics.

**Figure 1:**
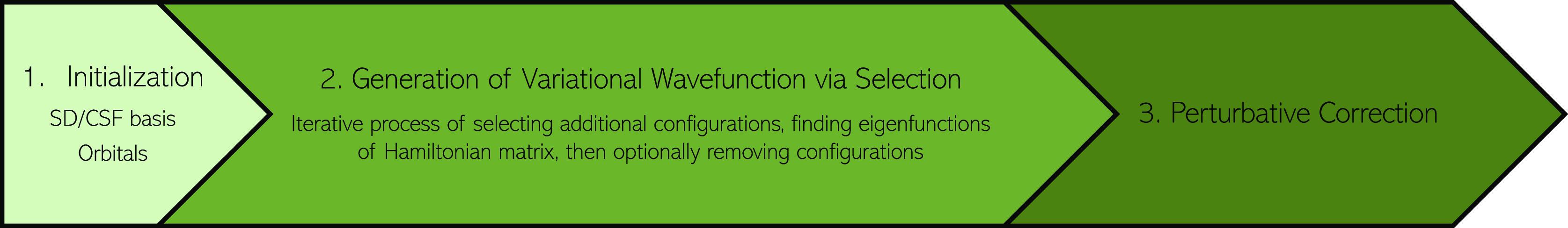
A simplified three-step schematic of a general selected configuration interaction algorithm.

Many different flavors of SCI theories exist,^32,38−47^ such as the coefficient-driven adaptive sampling CI (ASCI) approach,^33,48^ the stochastic Monte-Carlo CI (MCCI) approach,^28,49−52^ and the error-controlled adaptive CI (ACI)^34,35,53^ approach to name but a few, all essentially following the three-step methodology shown in Figure 1—initialization, generation of variational wave function via selection, and a perturbative correction to the energy.

In general, a SCI algorithm constructs a variational wave function (|Ψ_SCI_⟩) by iteratively building up the wave function via a specific selection criteria until self-convergence, normally of the energy (*E*_SCI_), is achieved. This iterative process permits the potential exploration of the complete FCI space as the calculation evolves, in contrast to truncated configuration interaction (CI) approaches, which limit the calculation to a given excitation or substitution level relative to a given reference. Optionally, to recover the small component of remaining dynamic correlation, *E*_SCI_ may be augmented by a nonvariational perturbation correction, usually in the Epstein–Nesbet (PT2) formalism to second order, resulting in *E*_SCI–PT2_. These methods are inherently black-box requiring very little user input, unlike the complete active space self-consistent field (CASSCF) approach,^54−57^ which utilizes FCI in a set of highly specific active orbitals that are adequate to describe the property of interest. It should be noted that in CASSCF both the orbitals and expansion coefficients are optimized simultaneously. In contrast, SCI approaches only perform an optimization of the latter and use a fixed set of orbitals, typically HF, although natural orbital schemes have also been implemented.^26,34^ SCI methods have also been employed as approximate CI solvers in CASSCF-like computations,^58,59^ allowing more flexible active spaces (*n*, ϕ ≈ 50) to be chosen, which is significantly larger than the current limit of CASSCF computations (*n*, ϕ = 20).^60^

It should be noted that sophisticated methods which bypass the need for explicit diagonalization of the Hamiltonian matrix exist, most notably the FCI quantum Monte Carlo (FCIQMC) approach.^61^ Within this approach, walkers explore the configurational space, and the expansion coefficient is taken to be proportional to the signed sum of walkers situated on each configuration after a large number of iterations; thus, walkers have sampled the space. However, in FCIQMC, and the initiator extension *i*-FCIQMC,^62^ care has to be taken to limit the degree of numerical noise, stemming from sign incoherence, and sampling bias.

Currently, the comparison and further development of SCI algorithms are difficult as these algorithms are implemented as standalone local in-house codes or (interfaced) in various electronic structure codes themselves. For example, the ACI method is implemented in FORTE^63^ which is a plugin to PSI4^64^ whereas the ASCI method is included in Q-CHEM.^65^ The MCCI method of Greer^66^ is implemented as a standalone code, relying on one- and two-electron integrals generated by MOLPRO,^67−69^ TURBOMOLE,^70^ or COLUMBUS.^71^ The heat-bath CI (HCI) method is implemented in the standalone code DICE,^72^ and as a module in PySCF.^73^ Additionally, ORCA^74^ and Quantum Package^45^ also have the capabilities of performing SCI calculations.

**Figure 2:**
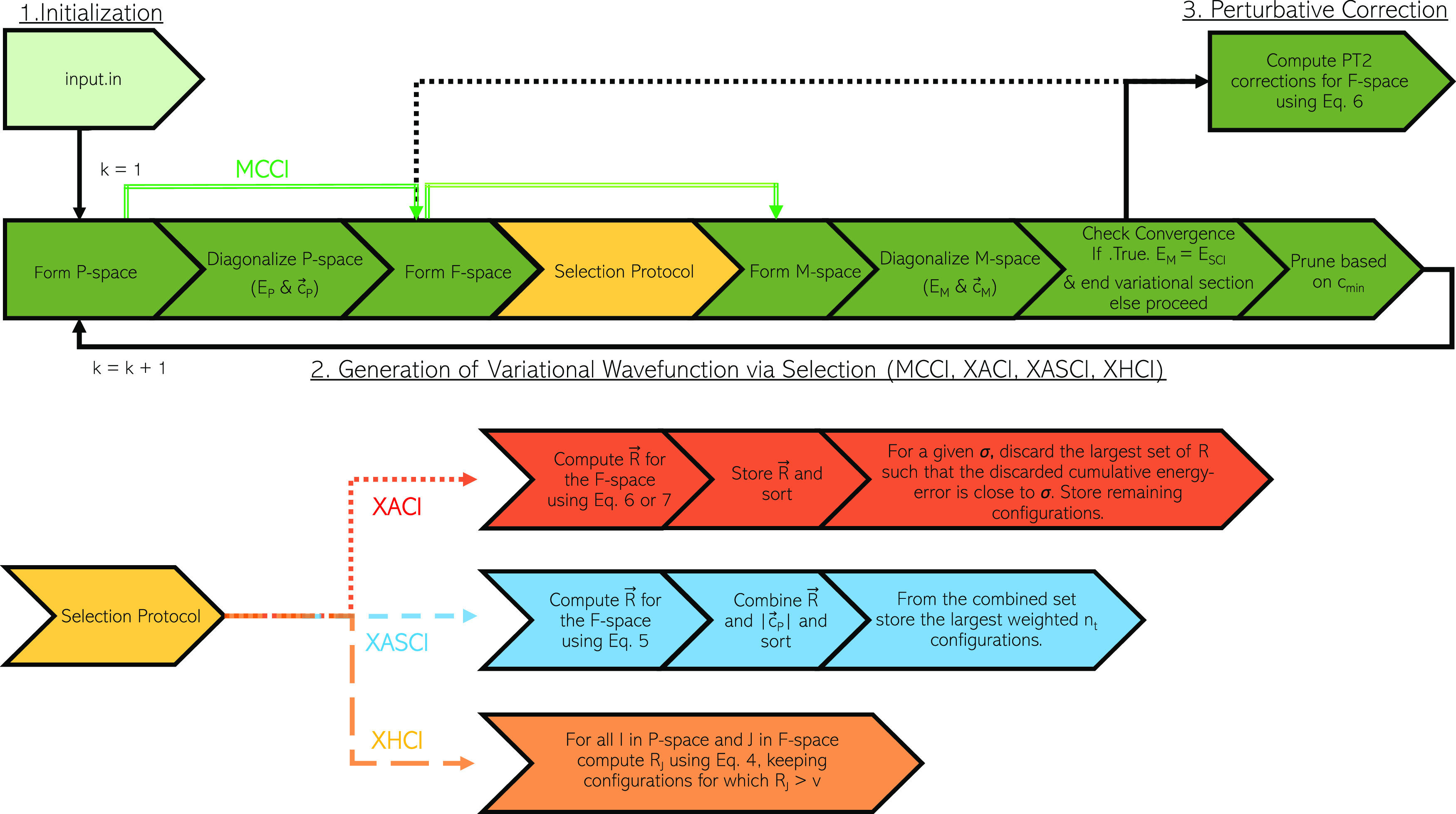
A schematic comparing the MCCI, XACI, XASCI, and XHCI algorithms.

In this work, we used the established framework of the MCCI code to develop a modular SCI code which is capable of employing various selection protocols to generate SCI wave functions. The code is developed such that common functionality is shared between the SCI algorithms, meaning that they only differ in which selection protocol subroutine is called (see Figure 2). We envisage that this approach will aid in the application of new selection processes. Additionally, as both SD and CSF bases are built into the MCCI code, we have the ability to choose which (*n*-electron) basis to use. To the best of our knowledge, for some of the algorithms discussed herein, this is the first time they are performed in a CSF basis.

The layout of this work is as follows. In Section 2, we begin by briefly discussing the main components of a general SCI algorithm and outline the background literature for the algorithms of interest, discussing in detail the main differences between various flavors of SCI algorithms. In Section 3, our implementation of the algorithms is discussed thoroughly. Section 4 gives details of the calculations with comparisons of computed PESs provided in Section 5, specifically the PESs of H_2_O, C_2_, and CH_4_ dissociation. Section 6 concludes this work and outlines the current research avenues.

## 2 Background

This section serves to provide a brief overview of the established literature for various SCI algorithms of interest and their underlying theories. For more detail, we encourage the reader to consult the cited work.

### 2.1 General Selected Configuration Interaction Algorithm

A general SCI wave function is constructed as a linear combination of SDs or CSFs, |Ψ_SCI_⟩ = *∑*_*I*_*c*_*I*_|ψ_*I*_⟩, subject to a specific selection routine, where *c*_*I*_ represents the expansion coefficient (importance) of the *I*th *n*-electron basis function (ψ_*I*_), commonly termed a configuration. For a given nuclear geometry, the molecular orbitals of the system are constructed. In this work, we primarily limit our discussion to HF orbitals; however, we also show that approximate natural orbitals can also be used.

The variational portion of a general SCI mechanism normally consists of an iterative process of augmentation and diagonalization with optional pruning to build |Ψ_SCI_⟩. At the start of each iteration, the reference space (*P*-space) is constructed, which on the first iteration could be the HF configuration or the set of configurations from a small CAS-CI calculation, CASSCF calculation without orbital optimization, and in future iterations would be configurations which remain from the preceding cycle. The augmentation step involves generating all, or a subset of, unique configurations that can be generated via single or double electron substitutions from the *P*-space, this space is commonly referred to as the interacting space (*F*-space) as it only contains configurations which can interact with a given *P*-space. Normally, a ranking function (*R*) is then assigned to each member of the *F*-space, which is specific to the SCI algorithm, and depending on the algorithm, either the *P*-space ∪ *F*-space or just the *F*-space is truncated, completing the augmentation step.

A matrix-eigenvalue equation, , is solved within the truncated space (*M*-space). Here, **H** and **S** represent the Hamiltonian and overlap matrices, respectively, and  represents the collective set of configuration expansion coefficients which minimize the energy (*E*_*M*_). In the SD basis, the matrix-eigenvalue equation reduces to  due to the orthonormal relationship between individual SDs (**S** = 1). If the wave function or energy has not yet converged to a suitable user-defined threshold, the *M*-space is taken to form the next *P*-space. Optionally, pruning of the wave function may also take place before the start of the next iteration. As we will see, various pruning algorithms are available,^33,34,51^ which aim to pass only the most important configurations to the subsequent *P*-space thus limiting the size of the *F*-space, which can become computationally demanding to generate and work through. After convergence has been achieved, the final variational energy, *E*_SCI_ = *E*_*M*_, may be augmented by a nonvariational correction.

### 2.2 Adaptive Sampling Configuration Interaction

The ASCI method was developed by Whaley and co-workers in 2016.^33^ For all the configurations (SD basis) in the *F*-space, *R* approximates, to the first order, the expansion coefficient (absolute) relative to the known *P*-space (see eq 1).
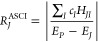
1Here, *H*_*IJ*_ refers to the individual Hamiltonian matrix element (HME) between configuration *I*, a member of the *P*-space, and configuration *J*, the trial configuration, with *E*_*P*_ and *E*_*J*_ representing the energy of the *P*-space and of configuration *J*, respectively. The former energy is obtained by solving the matrix-eigenvalue within the *P*-space, , and the latter is given by *H*_*JJ*_. At each iteration, the set of *R* values are combined with  and the largest *n*_t_ configurations are taken to form the *M*-space, where *n*_t_ is user-defined and limits the dimensionality of the variational wave function. In this work, it is the only method for which the size of the variational wave function is predetermined, approaching FCI limits as *n*_t_ tends toward the number of configurations in the FCI wave function. Unless convergence of the energy is achieved, the next *P*-space is taken to be the *n*_c_ configurations, where *n*_c_ is the second and final user-defined parameter with the largest absolute weighting. After convergence of the variational wave function has been achieved, the PT2 correction can be added to *E*_ASCI_(*n*_t_), giving *E*_ASCI-PT2_(*n*_t_). For the energy of the X^1^Σ_g_^+^ state of C_2_, it was shown that chemical accuracy, normally within 1 kcal/mol of the FCI energy, could be obtained for *E*_ASCI_(*n*_t_ = 2 × 10^5^).^33^ In the same study, the ASCI(*n*_t_ = 2 × 10^5^)-PT2 method was also found to predict the ground-state energy of the notorious Cr_2_ system with an accuracy comparable to that computed by the density matrix renormalization group (DMRG) algorithm. In a later study,^48^ the ASCI method was applied to the G1 test set, predicting atomization and ground-state energies to very high accuracy. Recently, ASCI has been used as the CI solver in CASSCF, allowing application to much larger active spaces than conventional CASSCF.^59^

### 2.3 Heat-Bath Configuration Interaction

The heat-bath configuration interaction (HCI) algorithm, developed by Umrigar and co-workers, uses a similar ranking function to that used in ASCI, with the exception that the energy difference in the denominator is no longer considered.^32^ Once again at each iteration the entire *F*-space is considered, and a configuration *J* (SD basis) is included in the *M*-space only if |*H*_*JI*_*c*_*I*_| for any *I* in the *P*-space is larger than ν, which is user-defined and in units of energy.

In the HCI algorithm, the restrictive *n*_t_ parameter is removed and the wave function is not normally pruned at the end of each iteration. It should be noted that one of the main benefits of the HCI algorithm is the heat-bath sampling algorithm, which is highly efficient as no time is wasted generating doubly excited determinants that are not added to the *M*-space. The authors achieve this by an additional setup step at the start of the algorithm which generates all of the HMEs corresponding to double excitations for each unique pair of orbitals, which is then sorted based on the absolute value in descending order. The authors take advantage of the fact that the HME for double excitations, up to a phase sign, is dependent only on the two-particle and two-hole orbitals involved in the excitation. Based on a given *P*-space, the *M*-space can then be generated by scanning through all the unique pairs of occupied orbitals on configuration *I*, generating all the configurations for which *H*_*IJ*_ is larger than , before moving onto the next *P*-space configuration. The lists are then merged, and duplicates are removed. In contrast, all singly excited determinants are tested as possible additions; however, the number of these determinants is greatly reduced when compared to the doubly excited space. A similar procedure is implemented when selecting configurations for the nonvariational PT2 correction. A semistochastic HCI algorithm has also been developed^41,43^ which aims to reduce the memory requirement of the nonvariational portion of the algorithm and has been applied to excited states,^36,37^ the PES of Cr_2_^75^ and as a CI solver in CASSCF.^58^ Extrapolations to the complete basis set limit have also been undertaken for small molecules^76^ and transition metals.^77^ The parallelization of the HCI algorithm has also been significantly improved allowing large active spaces (*n* = 22, ϕ = 168) to be considered.^78^ In our initial implementation of a HCI-selection-like algorithm, we use the standard approach of SCI theories and generate the entire *F*-space, subjecting them to HCI selection criteria.

### 2.4 Adaptive Configuration Interaction

The ACI algorithm was developed by Evangelista in 2014.^34,53^ At the heart of this approach is the user-defined parameter σ, σ ≈ |*E*_ACI_(σ) – *E*_FCI_|, which aims to provide a level of control of the energy-error with respect to FCI and is normally on the mHa energy scale. As σ decreases, the energy will tend toward the FCI limit, with the two equating for σ = 0 Ha. In this method, the *R* value assigned to each *J* in the *F*-space (SD basis) relates to the energy difference, or alternatively energy error, between the trial configuration *J* and the *P*-space, which can be predicted using PT2 or degeneracy corrected perturbation theory (DCPT)^79^ (see eqs 2 or 3, respectively).
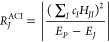
2
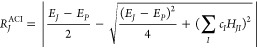
3

The error-controlled aspect of this algorithm is established by sequentially removing configurations, starting from the smallest *R*_*J*_, such that the cumulative energy error of the discarded configurations is approximately σ. The remaining configurations are added to the *P*-space, forming the *M*-space. If convergence is not achieved the wave function is pruned, based on a cumulative sum of , such that the cumulative sum of kept configurations, starting from the largest weighted, is smaller than 1 – γσ, where γ is the second user-defined parameter and has units of inverse energy. Additionally, the authors also suggest an approach where the nonvariational correction is approximated by adding the excluded *R*_*J*_ values to *E*_ACI_(σ), those which are eliminated when forming the final *M*-space, thereby eliminating the need to consider the entire *F*-space for the converged wave function.

In a dynamical sense, controlling the energy error between a series of points is of utmost importance as exact error cancellation would produce relative energies that are exact. For example, shifting the FCI predicted energies by a constant amount across an entire PES will result in a new PES with exactly the same shape, and thus a dynamical framework identical to FCI. Commonly, the nonparallelity error (NPE)^80^ is used as a quantitative measurement to determine the accuracy of an approximated PES relative to the most accurate PES available. The NPE is given by the difference between the maximum and minimum absolute errors across the PES. ACI has been shown to be able to predict the ground state singlet N_2_ dissociation surface with sub kcal/mol accuracy^34^ and further extended to excited states both in a state-specific and state-averaged regime.^35^ Additionally, ACI has been coupled to the density fitted second-order perturbative multireference-driven similarity renormalization group (DSRG-MRPT2) approach,^81−83^ to help recover the remaining dynamic correlation.^84^

### 2.5 Monte Carlo Configuration Interaction

All the variational SCI methods outlined previously are deterministic in nature; however, stochastic methods do exist. The MCCI algorithm developed at the turn of the century is one such example.^50−52^ In this algorithm, the *M*-space is formed by augmenting the *P*-space with a random subset of configurations from the *F*-space. This approach removes the need to generate the entire *F*-space and also does not require *R* values to be computed. After diagonalization of the *M*-space, the set of configurations with an absolute expansion coefficient greater than or equal to a user-defined threshold (*c*_min_) are kept, |*c*_*i*_| ≥ *c*_min_, and the process repeated until convergence. Previously MCCI has been applied to ground-state^27^ and state-averaged PESs including conical intersections^28^ and has been extended to calculate spin–orbit couplings,^85^ multipole moments,^28^ X-ray absorption,^86^ and scattering predictions.^87^ A systematic variant to MCCI (SMCCI) was also explored which utilizes the same selection criteria as MCCI but considers the entire *F*-space as a set of noninteracting batches, subjecting the *i*_add_ configurations with the largest weighting across all batches to the MCCI selection criteria.^38^ In a general sense, SMCCI can be considered to share similarities with ASCI where *i*_add_ is similar to *n*_t_, with the exception that the former is the maximum number of configurations that can be added per iteration. The selection criteria in each of the approaches differ as SMCCI explicitly diagonalizes many (small) Hamiltonian matrices per iteration, based on the *i*_batch_ parameter, whereas ASCI uses approximate expansion coefficients.

## 3 Implementation

All SCI algorithms were written in FORTRAN 90 using a locally adapted version of the existing MCCI V4 code.^52,66^ In this section, we outline our implementation of the ACI, ASCI, and HCI-adapted algorithms, which are herein labeled as XACI, XASCI, and XHCI, respectively. We do so to distinguish these methods from the established literature due to small differences in the algorithms. The algorithms are compared schematically in Figure 2.

### 3.1 Initialization

The first step of each calculation is the initialization step. For a given nuclear arrangement, the orbitals (restricted) are constructed and the one/two-electron integrals are generated. Throughout this work, we utilize the orbitals/integrals generated from MOLPRO via the DUMP argument to the FCI program. The resulting file (FCIDUMP) also includes the nuclear-repulsion energy.

Next, an input file is generated (mcci.in), which is needed by the SCI program. In this file, various parameters such as the number of α(*n*_α_)- and β(*n*_β_)-spin electrons, spin (*m*_s_) and spatial symmetry of the requested wave function, CSF or SD *n*-electron basis functions, frozen orbitals, number of requested roots (*i*_eig_), *c*_min_, convergence criteria, and various SCI dependent parameters (σ, *n*_t_, or ν) are provided by the user. If the calculation is started from a single configuration, then the occupied molecular orbitals (MOs) for each spin are also provided in this file. Alternatively, calculations may be started using a CI vector from a previous calculation. At this stage, any duplicates in the *P*-space are removed.

### 3.2 Generation of Variational Wavefunction via Selection

The variational portion of the SCI code starts by generating an initial *P*-space. Once the initial *P*-space has been determined, the matrix-eigenvalue equation is solved, storing the *P*-space and , collectively the *P*-space wave function (|Ψ_SCI_^*P*^⟩), and the resulting *E*_*P*_. Herein, all wave functions are normalized, , where *D*_norm_ represents ⟨Ψ|Ψ⟩. As per the Slater–Condon (Slater–Condon–Harris) rules, the HMEs between SDs (CSFs) which differ by more than two spatial orbitals will by necessity be zero. Therefore, all, nonidentical, spin-preserving configurations which differ in the occupation of one or two orbitals with respect to a given *P*-space are generated, which are stored and form the *F*-space. Using annihilation  and creation  operators, the entire *F*-space can be written as , where indices A and B (C and D) represent the set of occupied (unoccupied) orbitals and B > A (D > C). Additionally, the generated configurations also conserve the overall spatial symmetry. This means for single excitations the occupied and virtual orbitals must belong to the same irreducible representation (irrep), irrep(A) = irrep(C), whereas for double excitations the product of the irreducible representation of the occupied orbitals must match that of the virtual orbitals, irrep(A) × irrep(B) = irrep(C) × irrep(D). In MCCI, only a stochastic subset of the *F*-space is generated. For CSFs, we first generate all SDs in the *F*-space without removing duplicates. Then for each SD we use the approach within MCCI of a random walk through the branching diagram^51^ to give lists of spin orbitals corresponding to linearly independent CSFs, once duplicate lists are removed. As the path through the branching diagram is stochastic, then it is possible that some CSFs are missed. However, we expect the fraction to be small as we start with many more SDs than there are CSFs.

The next stage of the algorithm is determining *R* for each trial configuration, collectively , which as mentioned previously is specific to the SCI algorithm. As not only the *R* function differs between SCI methods but also the selection procedure, we discuss the entire selection protocol in sequence starting with the XHCI algorithm. Additionally, the matrix elements between configurations are computed on the fly to avoid having to store them as the Hamiltonian and overlap matrices in the current set of configurations are stored and then modified with new elements in MCCI V4.

The original HCI algorithm was developed to work in the SD basis, therefore we updated the ranking formula (see eq 4) to accommodate both *n*-electron bases, where *S*_*JI*_ represents the overlap matrix element (OME) for configuration *I* and *J*. We use a similar approach to ref (26) to take into account that nonorthogonal CSFs are employed. For the trial configuration *J* to be included in the *M*-space, the condition *R*_*J*_^HCI^ > ν must be satisfied for at least one *I* in the *P*-space, as mentioned previously ν has units of energy. Normalization of the trial configuration by  is performed, this is also done for the other SCI algorithms. In this approach, no storage or sorting of  is required. After exhausting the *F*-space the set of configurations which fulfill this condition are combined with the *P*-space, with this combined set forming the *M*-space.
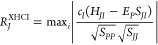
4

We now turn our attention to the XASCI selection protocol, which uses an updated ranking formula (see eq 5). In this approach  is stored and combined with . This combined set is then sorted in descending order, where the largest-weighted *n*_t_ configurations form the *M*-space. Sorting is performed by the quick-sort algorithm. Additionally, if the number of configurations in the *P*-space ∪ *F*-space is less than *n*_t_, which can happen in early iterations or for large *c*_min_ values, then no configurations are omitted.
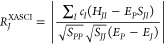
5

The final selection protocol outlined is that utilized by the XACI algorithm. Analogous to the original ACI algorithm, we have the ability to use both PT2 or DCPT to predict each *R*_*J*_ (see eqs 6 or 7 for the updated formulas, respectively). In this work, all XACI calculations use the DCPT formalism. After exhausting the entire *F*-space,  is sorted in descending order, once again using the quick-sort algorithm. The selection criterion used in this algorithm is analogous to the cumulative sum used in the original ACI algorithm and is governed by the energy-error threshold, σ, which as mentioned previously is in units of energy. Beginning from the configuration with the smallest *R*_*J*_, configurations are discarded until the cumulative sum condition is satisfied, i.e., the energy-error of the discarded set is close to . The remaining configurations are combined with the *P*-space and form the *M*-space.
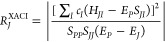
6
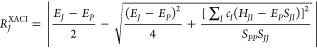
7

After defining the *M*-space, the matrix-eigenvalue equation is solved, giving |Ψ_SCI_^*M*^⟩ and *E*_*M*_. At this point, *E*_*M*_ is checked for convergence, which is taken to be the iteration at which the absolute energy difference with respect to the previous iteration is less than 10^–5^ Ha. For MCCI, the standard convergence routine outlined in ref (38) was used. If convergence has been achieved, the variational portion of the calculation will conclude, giving the final SCI wave function, |Ψ_SCI_⟩ = |Ψ_SCI_^*M*^⟩, and *E*_SCI_. Alternatively, if convergence has not yet been achieved, the iteration finishes with a pruning step. The original ACI and ASCI algorithms have their own pruning protocols governed by 1 – γσ and *n*_c_, respectively, which we have implemented for our adaptations. However, in this work, XACI, XASCI, and XHCI algorithms use the MCCI selection criteria to prune the wave function between iterations. For a configuration to survive, the absolute expansion coefficient must be larger than *c*_min_, normally this is on the order of 10^–3^ to 10^–4^. All configurations would survive when a *c*_min_ value of 0 is used. For CSFs, we transform the coefficients to an approximate orthonormal basis, see ref (88) for further details. In this transformation, *c*_min_ is scaled by *∑*_*I*_*c*_*I*_*S*_*II*_*c*_*I*_ and the nonorthogonal coefficient is scaled by  (see eq 8). This pruning equation also holds for SDs as the orthonormality condition ensures that *S*_*II*_ and *∑*_*I*_*c*_*I*_*c*_*I*_ reduce to unity, thereby comparing  directly to *c*_min_.
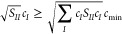
8

After pruning has completed, the resulting configurations form the subsequent *P*-space and the calculation proceeds iteratively until convergence has been achieved.

### 3.3 Non-Variational Correction

In order to account for more of the remaining dynamic correlation, a nonvariational correction to the variational energy may be required. If this is the case, the *P*-space is taken to be the configurations which constitute |Ψ_SCI_⟩ and the PT2 corrections are computed for each *J* in the *F*-space (using eq 6), where *E*_*P*_ = *E*_SCI_. The individual corrections are then added to *E*_SCI_. For consistency we also use this scheme to compute the PT2 correction for MCCI instead of the MCCI-PT2 regime outlined in ref (26).

### 3.4 Spin Adaptation

**Figure 3:**
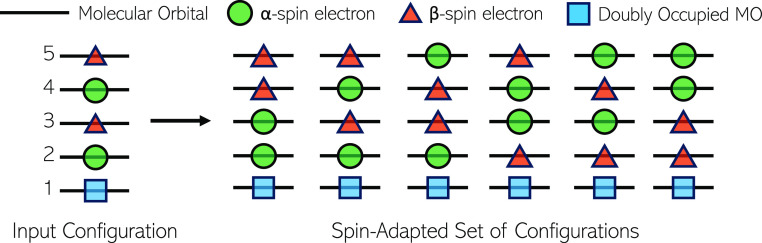
A schematic showing the spin-adapted set of configurations that can be generated for a given input configuration.

Wave functions consisting of SDs are strictly only pure eigenfunctions of the  operator and not necessarily the  operator. For example, in a two-electron system, the *m*_s_ = 0 component of a singlet and triplet state could mix and produce spin-impure wave functions. This mixing would become more prevalent as the energies of different spin manifolds, which share a common *m*_s_, become close. This would not be an issue for wave functions consisting of CSFs as these, by construction, are eigenfunctions of both  and . To aid convergence to spin-pure states, we implemented the spin-adaptation regime of ref (89), see the cited literature for an in-depth overview as only a brief outline follows. First, for a given wave function, the number of unique molecular orbital occupations are generated. In this nomenclature, the configuration 1α 1β 2α 3β 4α 5β on the left-hand side of Figure 3 would reduce to the MO occupation vector (2, 1, 1, 1, 1). Second, for each unique MO occupation vector the number of MOs containing a single electron are identified; if all orbitals are doubly occupied, then no further action is required for the configuration. For those containing singly occupied orbitals the set of configurations which can be built from all permutations of the unpaired electrons in the singly occupied orbitals, for a fixed *m*_s_, are generated and then recombined with the doubly occupied orbitals (see Figure 3). When required, the spin-adaptation procedure is carried out prior to diagonalization of the *P* and *M*-space, thus ensuring these spaces are spin complete.

### 3.5 State-Averaged Approach

Until this point, we have only discussed state-specific (SS) regimes, whereby a set of configurations is optimal for a single root. However, we have also implemented state-averaged (SA) approaches for each of the algorithms in which a single configurational space now describes the lowest-lying *i*_eig_ roots. Extending the algorithms requires reconsideration of various portions of the SCI algorithms, such as calculating  and the way in which the wave function is pruned. Additionally, if *i*_eig_ > 1 the Davidson–Liu diagonalization routine is used as *i*_eig_ eigenvectors can be returned simultaneously. We will briefly discuss the changes when working in a SA framework. It should be noted that ACI and HCI have been implemented in a SA fashion similar to what follows.^35,36^ The idea of SA-ASCI has also been explored previously.^33^

In SA-XACI and SA-XASCI each *R*_*J*_ value is computed with respect to each of the *i*_eig_ roots , where  and  are used, and *i*_root_ ranges from 0 to *i*_eig_ – 1. In this approach, the number of individual *R*_*J*_ values to be computed increases by a factor of *i*_eig_. For each trial configuration, the average *R*_*J*_ value is stored in  (see eq 9) and subjected to the selection criteria. For XASCI, the average of  across all roots is also used and stored in . In XHCI, no averages are computed; instead, inclusion into the *M*-space will happen if the selection condition is fulfilled by at least one root.
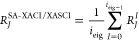
9

In terms of the pruning regime, we use the SA-MCCI approach.^28^ In this, the cumulative coefficient (eq 10) is instead tested against *c*_min_. Using this cumulative approach will always keep more configurations than subjecting the individual coefficients. To aid convergence, on the first iteration we use the SS algorithm switching to a SA regime for each subsequent iteration. Additionally, this allows the HF configuration to be used as the initial *P*-space in SA calculations.
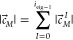
10

The convergence criterion used was also updated. For each *i*_eig_, the energy difference between the current and previous iteration is computed, which is then averaged. The absolute value is then tested. Once again, MCCI uses a different criteria to test for convergence.

### 3.6 Parallelization

The algorithms can be run either in serial or in parallel, with the latter communicating through a message passage interface (MPI). The only portion of the code that is run in parallel is the prediction of the ranking functions, as these are uncoupled and rely only on each process having the current *P*-space. Therefore, this can be made embarrassingly parallel, as each can be computed independently.

The calculation begins with each process receiving the initial *P*-space, which is subsequently diagonalized. The rank 0 process (head core) then generates the *F*-space and shares this equally and systematically among all available processes. The remainder is retained on the head core. For XACI and XASCI, each process computes their subset of  that is passed back to the rank 0 process. On this head core, the *M*-space is then constructed and diagonalized and, if required, pruned. Prior to the start of the next iteration, the remaining configurations are shared so that each process had the same *P*-space. In XHCI, parallelization works in a slightly different manner. In this method each process keeps track of the configurations which fulfill the selection criteria, passing them back to process 0. The iteration proceeds in an analogous fashion to that of XACI and XASCI. The constructed XACI, XASCI, or XHCI wave functions are independent of the number of available processes. However, this is not necessarily the case for MCCI. In MCCI each process generates a random portion of the *F*-space and tests this set of configurations for addition to the wave function. The configurations remaining on each process are then shared with all others. Therefore, a large number of cores helps MCCI to find more possible additions. All calculations were run across 12 cores.

## 4 Computational Details

When referring to a particular SCI algorithm, we use the following notation, SCI(*Y*,*Z*) where *Y* refers to either σ (kcal/mol), *n*_t_ (SDs/CSFs) or ν (kcal/mol), and *Z* refers to the *c*_min_ value. As MCCI relies solely on *c*_min_ only, this value is provided.

All SCI calculations were performed using a developmental version of GeneralSCI 1.0 which is freely available at ref (90). The orbitals and one/two-electron integrals were generated by MOLPRO 2015.1.^67,91^ When comparison is made to FCI values calculated herein, we used either the FCI program in MOLPRO 2015.1^92,93^ (H_2_O and CH_4_) or the DETCI module in PSI4 (C_2_).^64,94^ For coupled cluster (CC) and multiconfigurational self-consistent field (MCSCF) calculations, we used MOLPRO 2015.1, specifically the closed-shell CC^95,96^ or MULTI^97,98^/RS2^99^ programs, respectively. Comparisons to available literature data are also made.

The first PES that was explored corresponds to the symmetric double-hydrogen dissociation in H_2_O for various spin manifolds. The oxygen-hydrogen distance (rOH) was varied from 1.51 to 5.67 a_0_ maintaining *C*_2*v*_ spatial symmetry across all points. The HOH angle was fixed at 104° and the spherical cc-pVDZ basis set was used, invoking the frozen-core approximation for the low-energy 1s-like orbital (1a_1_) of oxygen (*n* = 8, ϕ = 23). We focus on three specific spin states, the singlet (^1^A_1_) state, *n*_α_ = *n*_β_ = 5, the triplet (^3^A_1_) state, *n*_α_ = 6 and *n*_β_ = 4, and finally the quintet (^5^A_1_) state, *n*_α_ = 7 and *n*_β_ = 3. All H_2_O SCI calculations were run in a SS fashion and use the ^1^A_1_ RHF orbitals, with the exception of the natural orbital comparison which use natural orbitals obtained from MCCI. Additionally, the initial *P*-space for the SCI calculations was generated by running a CAS-CI(8,7) calculation where the active space consisted of the valence 2a_1_, 3a_1_, 4a_1_, 1b_1_, 1b_2_, 2b_3_, and 3b_2_ orbitals. The same valence active space was used in the MCSCF calculations.

The dissociation of C_2_ was then explored in an SA fashion by the various SCI algorithms. The distance between the carbon atoms (rCC) was varied from 2.08 to 3.78 a_0_. All C_2_ computations were performed within the largest Abelian point group *D*_2*h*_, instead of the true nonabelian point group of the molecule *D*_∞*h*_, in which the states of interest, namely ^1^Σ_g_^+^ and ^1^Δ_g_, transform with A_g_ symmetry. The Cartesian 6-31G* basis set was used in all computations with the core 1σ_g_ and 1σ_u_ orbitals doubly occupied in all configurations (*n* = 8, ϕ = 28). We use these orbital labels instead of the irreducible representation within the *D*_2*h*_ point group. The initial *P*-space for the SCI algorithms was once again generated from a CAS-CI(8,8) space involving all the valence orbitals, 2σ_g_, 2σ_u_, 1π_u(*x*)_, 1π_u(*y*)_, 1π_g(*x*)_, 1π_g(*y*)_, 3σ_g_, and 3σ_u_. In the majority of the calculations, the first iteration is run in an SS fashion (*i*_eig_ = 1) considering only the ground state, increasing to a SA regime (*i*_eig_ = 2) for all other iterations. However, at an rCC of 2.27 a_0_, the ^1^Δ_g_ and the first excited ^1^Σ_g_^+^ states are only separated by 3.57 kcal/mol, which in some cases, resulted in the convergence of the SCI wave function to two ^1^Σ_g_^+^ states. For these anomalous cases, the first diagonalization of the *P*-space was instead solved for two roots, which aided convergence to the states of interest.

The final system of interest is the dissociation of a single hydrogen atom in CH_4_. The *C*_*s*_ point group was maintained throughout the entirety of the PES, stretching one hydrogen–carbon bond (rCH) from 1.8 to 7.18 a_0_. All other carbon-hydrogen bonds were fixed at 2.079 a_0_ in addition to freezing all HCH angles at 109.47°. The 1s-like orbital (a′) of carbon remained doubly occupied in all configurations and the cc-pVDZ basis was used (*n* = 8, ϕ = 33). The initial *P*-space used in the SCI calculations is solely the HF configuration.

To provide a quantitative measurement of the accuracy of an approximated PES, we consider both the NPE, described earlier, and the standard deviation (σ_SD_, see eq 11) of the energy difference with respect to FCI (ΔFCI), where ΔFCI = *E*_SCI(-PT2)_ – *E*_FCI_.^26^ Here, *I* ranges across all *M* points considered on the PES and  is the averaged energy difference given by . It should be noted that σ still refers to the ACI selection criterion whereas σ_SD_ refers to the standard deviation of ΔFCI.
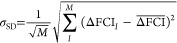
11

Comparison of the averaged absolute energy difference , is also explored across the methods. We use  as *E*_SCI-PT2_ can drop below *E*_FCI_ therefore it is entirely possible that situations could arise when ΔFCI = 0, due to the sign cancellation of errors, despite observed differences. Finally, depending on the *n*-electron basis functions employed,  or  is used to represent the number of configurations in Ψ_SCI_, once again averaged across the PES.

## 5 Results and Discussion

### 5.1 H_2_O Dissociation

**Figure 4:**
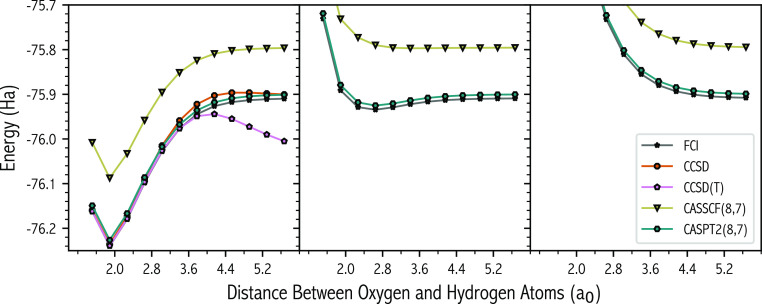
Energy of the ^1^A_1_ (left panel), ^3^A_1_ (center panel), and ^5^A_1_ (right panel) states as a function of both oxygen-hydrogen distances calculated using various electronic-structure theories. The legend applies to all panels. For the active space used, refer to the main text (Section 4) or Table S1.

The FCI surfaces of the ^1^A_1_, ^3^A_1_, and ^5^A_1_ states as a function of rOH are given in Figure 4, along with those predicted by standard electronic-structure theories, namely CC and MCSCF. The ^1^A_1_ and ^3^A_1_ states have a shape akin to a standard Morse potential, the latter has a smaller dissociation energy, 206.97 versus 15.86 kcal/mol, with the minimum shifted by around 0.8 a_0_. On the other hand, the ^5^A_1_ state is purely repulsive as rOH increases. The ^1^A_1_, ^3^A_1_ and ^5^A_1_ FCI wave functions in cc-pVDZ contain approximately 1.96 × 10^7^, 1.49 × 10^7^ and 6.38 × 10^6^ SDs, respectively, and become degenerate at large separations, energies are within 1.33 kcal/mol at an rOH of 5.67 a_0_. Previously, we have investigated the bond breaking ^1^A_1_ pathway using SD-MCCI(10^–3^) and SD-SMCCI, albeit a HOH angle of 104.5° was used.^38^ In this previous study, NPEs of around 5 and 6 kcal/mol were observed.

All SCI methods have the ability to generate extremely high-accuracy PESs, to sub kcal/mol accuracy, for the various spin states while using only a fraction of the FCI space (see Table 1 for a condensed comparison of selected methods/criteria or Table S1 for the complete version). In the interest of readability, in the text, the SS prefix is assumed when naming each of the methods. To note, in the CSF basis, the largest  was 136,145 CSFs [XHCI(ν = 0.06, 10^–4^), ^3^A_1_] which equates to approximately 0.9% of the FCI space (NPE and σ_SD_ of 0.15 and 0.05 kcal/mol, respectively), albeit the FCI space was in SDs. In contrast, the smallest  was as little as 149 [XACI(σ = 31.38, 10^–4^), ^5^A_1_] which equates to approximately 2 × 10^–3^% of the FCI space (NPE and σ_SD_ of 1.84 and 0.50 kcal/mol, respectively). As expected, tightening the selection criteria in each method improves the agreement to FCI, however, the convergence to a given accuracy is varied between the SCI selection protocols.

**Table 1:** Nonparallelity Error (NPE) of the Double-Hydrogen Dissociation Potential Energy Surfaces in H_2_O for Various Spin Manifolds and SCI Algorithms^a^

	^1^A_1_ state		^3^A_1_ state		^5^A_1_ state	
algorithm	NPE	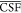	NPE	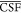	NPE	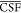
CSF-SS-XACI (σ = 31.18, 10^–^^4^)	5.99/6.29	227	11.95/10.66	349	1.84/1.60	149
CSF-SS-XACI (σ = 6.28, 10^–^^4^)	0.77/0.75	950	3.17/2.64	2071	0.97/1.02	986
CSF-SS-XACI (σ = 0.63, 10^–^^4^)	0.14/0.12	14,375	0.24/0.21	27,713	0.11/0.10	11,678
CSF-SS-MCCI (10^–3^)	3.48/1.08	1287	2.91/1.56	1853	5.54/0.36	1315
CSF-SS-MCCI (10^–4^)	0.65/0.24	16,526	0.69/0.16	19,330	0.68/0.02	12,401
CSF-SS-XASCI (*n*_t_ = 10^3^, 10^–^^4^)	6.05/1.69	1000	10.17/3.23	1000	12.52/1.35	1000
CSF-SS-XASCI (*n*_t_ = 10^4^, 10^–^^4^)	1.99/0.61	10,000	2.22/0.29	10,000	1.72/0.16	10,000
CSF-SS-XASCI (*n*_t_ = 5 × 10^4^, 10^–^^4^)	0.44/0.17	50,000	0.64/0.09	50,000	0.35/0.04	50,000
CSF-SS-XHCI (ν = 0.63, 10^–^^4^)	1.86/0.62	9048	2.28/0.33	11,634	1.53/0.09	7147
CSF-SS-XHCI (ν = 0.31, 10^–^^4^)	0.90/0.30	21,559	1.23/0.19	27,321	0.89/0.02	15,765
CSF-SS-XHCI (ν = 0.06, 10^–^^4^)	0.18/0.07	87,223	0.20/0.01	136,145	0.15/0.00	76,051
CSF-SS-XASCI (adaptive-*n*_t_, 10^–^^4^)	1.59/0.78	950				

a The properties relating to the variational and non-variational energies are separated by /. All NPEs provided are in terms of kcal/mol, and  refers to the surface-averaged dimensionality of the variational wavefunction.

**Figure 5:**
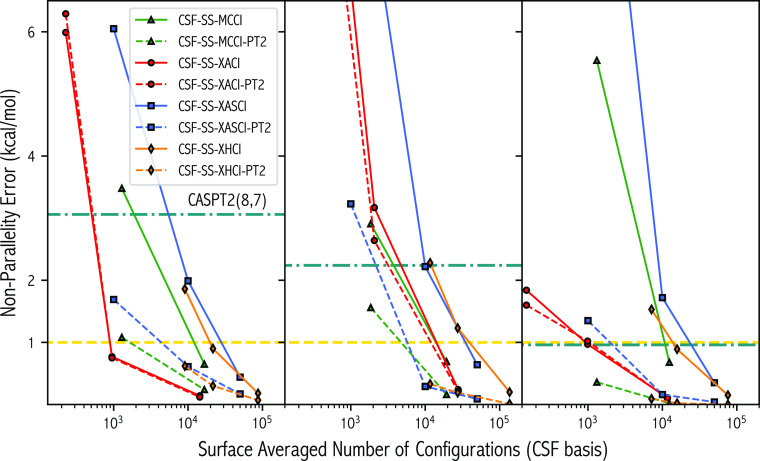
Nonparallelity error of the ^1^A_1_ (left panel), ^3^A_1_ (center panel), and ^5^A_1_ (right panel) states as a function of the surface-averaged dimensionality of the converged variational wave function for various selected configuration algorithms and selection thresholds.

For this PES and purely from a dimensionality-to-accuracy viewpoint, the selection protocol used in the XACI algorithm appears to be the best performer across the various spin states when considering the NPE of the variational energy. This can be seen clearly, especially for the ^1^A_1_ and ^5^A_1_ states, in Figure 5, which gives the NPE for each SCI approximated PES at a range of user-defined thresholds as a function of  on a logarithmic scale. For example, the CSF-XACI(σ = 0.63, 10^–4^) ^1^A_1_ surface has an NPE of 0.14 kcal/mol (σ_SD_ = 0.04 kcal/mol and ) for a  of 11,678 CSFs. Despite CSF-MCCI(10^–4^) having a similar  at 12,553 CSFs and a smaller  at 0.78 kcal/mol, the NPE is larger (0.65 kcal/mol) as consistent error control along the PES is more beneficial than aiming for FCI quality energies, i.e., small , when considering surface accuracy. Similarly, CSF-XHCI(ν = 0.31, 10^–4^) has a larger NPE (0.90 kcal/mol) despite a  of 15,765 CSFs. A comparable NPE (0.18 kcal/mol) is observed for CSF-XHCI(ν = 0.06, 10^–4^) but at the expense of  increasing drastically to 87,223 CSFs [7.5 times that of CSF-XACI(σ = 0.63, 10^–4^)]. For CSF-XASCI(*n*_t_ = 5 × 10^4^, 10^–4^), an NPE of 0.44 kcal/mol is observed which again fails to outperform CSF-XACI(σ = 0.63, 10^–4^) despite  being 4.3 times that of CSF-XACI(σ = 0.63, 10^–4^). For the ^1^A_1_ and ^5^A_1_ surfaces, CSF-XACI(σ = 6.28, 10^–4^), which corresponds to increasing σ by an order of magnitude, predicts NPEs within chemical accuracy (±1 kcal/mol) for a  of less than 1000 CSFs. This highlights the strength of the error-selection protocol and the highly compact yet highly accurate wave functions that can be obtained. For these spin states, no other method reaches this level of size-to-accuracy ratio. Interestingly, for ^3^A_1_, the NPE/σ_SD_ of the CSF-MCCI(10^–3^) (2.91/0.81 kcal/mol) surface is slightly smaller than that predicted by CSF-XACI(σ = 6.28, 10^–4^) (3.17/1.07 kcal/mol) despite  being 218 CSFs less, approximately a 10% reduction. At the tighter thresholds, the order is reversed with both NPEs being within chemical accuracy. This shows that for some systems, the stochastic MCCI approach can generate compact yet accurate wave functions which are competitive to XACI while XASCI and XHCI once again fail to reach this cost-to-accuracy ratio.

From an alternative viewpoint, the XASCI algorithm can be thought of as an approximation of the best variational wave function for a given *n*_t_, the approximation being that the selection criteria use coefficients determined by first-order perturbation theory (see eq 5) for configurations in the *F*-space. To find the best possible *n*_t_ configurations would entail considering all possible combinations of the *F*-space configurations with a given *P*-space which would generally be computationally intractable. This was similar to previous work undertaken by us in ref (38). To explore this viewpoint, we recomputed the ^1^A_1_ surface using CSF-XASCI and an adaptive *n*_t_ selection criteria, where *n*_t_ matches the number of CSFs in the CSF-XACI(σ = 6.28, 10^–4^) wave function. Indeed, for CSF-XASCI(adaptive-*n*_t_, 10^–4^) the  was lowered, highlighting a small improvement of the variational wave function in terms of  for the same . Despite this improvement, CSF-XACI(σ = 6.28, 10^–4^) still predicts a more accurate surface, albeit the CSF-XASCI(adaptive-*n*_t_, 10^–4^) surface is now far more competitive than the static CSF-XASCI(*n*_t_ = 10^3^, 10^–4^). However, the optimal a priori adaption of *n*_t_ for small NPEs and compact wave functions would be unknown. Additionally, the XHCI algorithm can also be considered as approximating the best variational energy this time for a given energy threshold (eq 4). Despite XACI also being based on an energy criterion the selection process is different which leads to the very different behavior of the methods.

Inclusion of the PT2 correction significantly lowers , NPE, and σ_SD_ for MCCI, XASCI, and XHCI, which is greatest for small . For example, the NPE of the XASCI(*n*_t_ = 10^4^, 10^–4^) and XHCI(ν = 0.63, 10^–4^) ^3^A_1_ surfaces lowers by almost 2 kcal/mol when including the nonvariational correction, resulting in FCI quality surfaces for modest wave functions. As a result of the error control in the XACI selection procedure, the NPE for a given σ remains practically unchanged between CSF-XACI-PT2 and CSF-XACI, the largest deviation between variational and nonvariational XACI NPEs was found to be approximately 0.5 kcal/mol. This is a highly desirable feature as computing the PT2 correction for large variational wave functions can be computationally demanding and time-consuming. For the XACI algorithm, the only noticeable difference between variational and nonvariational surfaces is in , which is much reduced in the later, and as explored earlier, does not necessarily influence the accuracy of the surface.

It should be stressed that the comparisons made solely rely on the relationship between dimensionality and energy and not on the overall timings of the SCI methods, which may outweigh the reduced dimensionality of a particular SCI method. However, in our initial implementation, optimality of the SCI algorithms in terms of speed was not considered in great detail.

For the tightest threshold parameters, the surfaces were recomputed in the SD basis. With the exception of the XHCI(ν = 0.06, 10^–4^) ^3^A_1_ and ^5^A_1_ surfaces, and the XASCI surfaces which by construction would be the same,  was larger than  for the same selection threshold. The largest SD-to-CSF ratios () were observed for the ^1^A_1_ surface, decreasing for the ^3^A_1_ and ^5^A_1_ surfaces in a stepwise fashion (see Figure S1), thus meaning that the reduced dimensionality when using a CSF basis was greatest for the ^1^A_1_ surface. It should be remembered that we are selecting in either the SD or CSF basis rather than giving the SDs corresponding to a given set of CSFs. For the ^1^A_1_ surface, the SD-to-CSF ratio was largest for the XACI methods (approximately 3.5:1) and was lower for MCCI and XHCI (approximately 1.5:1). The NPEs in each basis were also similar, with the largest deviation obtained for XASCI(*n*_t_ = 5 × 10^4^, 10^–4^) which differed by around 0.6 kcal/mol. Interestingly, in the SD basis,  for XACI(σ = 0.63, 10^–4^) is closer to σ when compared to the CSF counterpart (0.94 and 0.63 kcal/mol for ^1^A_1_, respectively). This became even more pronounced when the SD surface was recomputed with SD-XACI(σ = 6.28, 10^–4^). Additionally, the ^3^A_1_ surface predicted by SD-XACI(σ = 6.28, 10^–4^) was drastically improved in the SD basis, NPE reduced from 3.17 to 0.39 kcal/mol ( = 2348 SDs and  = 2071 CSFs).

The multireference (MR) measure (ζ_MR_)^29,100^ (see eq 12) was used to quantify the MR character of the wave functions in the SD basis. The closer ζ_MR_ approaches 1, the more MR the wave function is, with an ζ_MR_ of 0 signifying one configuration.
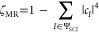
12

As is shown in Figure S2, for the SD-XACI(σ = 6.28, 10^–4^) ^1^A_1_ and ^3^A_1_ surfaces, ζ_MR_ increases drastically as a function of rOH, the former is slightly more MR at dissociation and the latter has a larger ζ_MR_ at small and intermediate rOH. In terms of the ^5^A_1_ state, the opposite is observed as ζ_MR_ decreases as a function of rOH. For all the SCI algorithms, the profile of ζ_MR_ for each of the spin manifolds was very similar. To verify that the wave functions had converged to the correct spin manifold,  was computed as described in ref (101). For large rOH, the SD-MCCI(10^–4^) and SD-XACI(σ = 6.28, 10^–4^) ^1^A_1_ wave functions were found to be spin contaminated, all other wave functions were found to be very close to 0, 2, or 6. Therefore, the SD-XACI(σ = 6.28, 10^–4^) ^1^A_1_ surface was recomputed, this time implementing the spin-adaptation procedure outlined earlier. This procedure eliminated any spin contamination (see Figure S2) at the expense of a greater , increasing by almost 70% (see Figure S1).

To investigate the effect of orbital choice on the dimensionality and energy of SCI wave functions, we compared energies and wave functions in the RHF and approximate natural orbital (NO) basis. We focus on a single point on the ^1^A_1_ surface (rOH = 4.54 a_0_) which shows a great deal of MR character and thus should benefit most from the approximate NOs. The approximate NOs used in the SCI calculations were generated from a SD-MCCI(10^–3^) calculation based on the implementation outlined in ref (26). The energy and dimensionality of selected SCI algorithms in both orbital bases are shown in Table 2, with the complete set shown in Table S2. In these SCI calculations, we revert to the CSF basis. Indeed, using the approximate NO basis greatly reduces the dimensionality of the SCI wave functions, except for XASCI due to the fixed wave function selection protocol. For MCCI, XHCI, and XASCI, we also observe a reduced ΔFCI when transitioning to the more optimal NO basis. For XACI, a similar ΔFCI in both basis is predicted, which results from the control of the energy error and thus should be hardly affected by the choice of orbitals.

**Table 2:** Energy Difference, with Respect to Full Configuration Interaction (ΔFCI), and the Dimensionality of the Variational Wavefunction (CSF Basis) Predicted in the Canonical Restricted Hartree–Fock or Approximate Natural Orbital Basis for Various Selected Configuration Interaction Algorithms^a^

	RHF orbitals		MCCI (10^–3^) NOs	
algorithm	ΔFCI	CSFs	ΔFCI	CSFs
CSF-SS-MCCI (10^–4^)	0.65	13,182	0.43	7168
CSF-SS-XACI (σ = 0.63, 10^–^^4^)	0.91	8553	0.88	3187
CSF-SS-XASCI (*n*_t_ = 10^4^, 10^–^^4^)	0.81	10,000	0.18	10,000
CSF-SS-XHCI (ν = 0.63, 10^–^^4^)	1.52	8472	0.60	6246

a All energies are given in terms of kcal/mol. Both oxygen-hydrogen distances were set to 4.54 a_0_ and only the ^1^A_1_ state is considered.

### 5.2 C_2_ Dissociation

**Figure 6:**
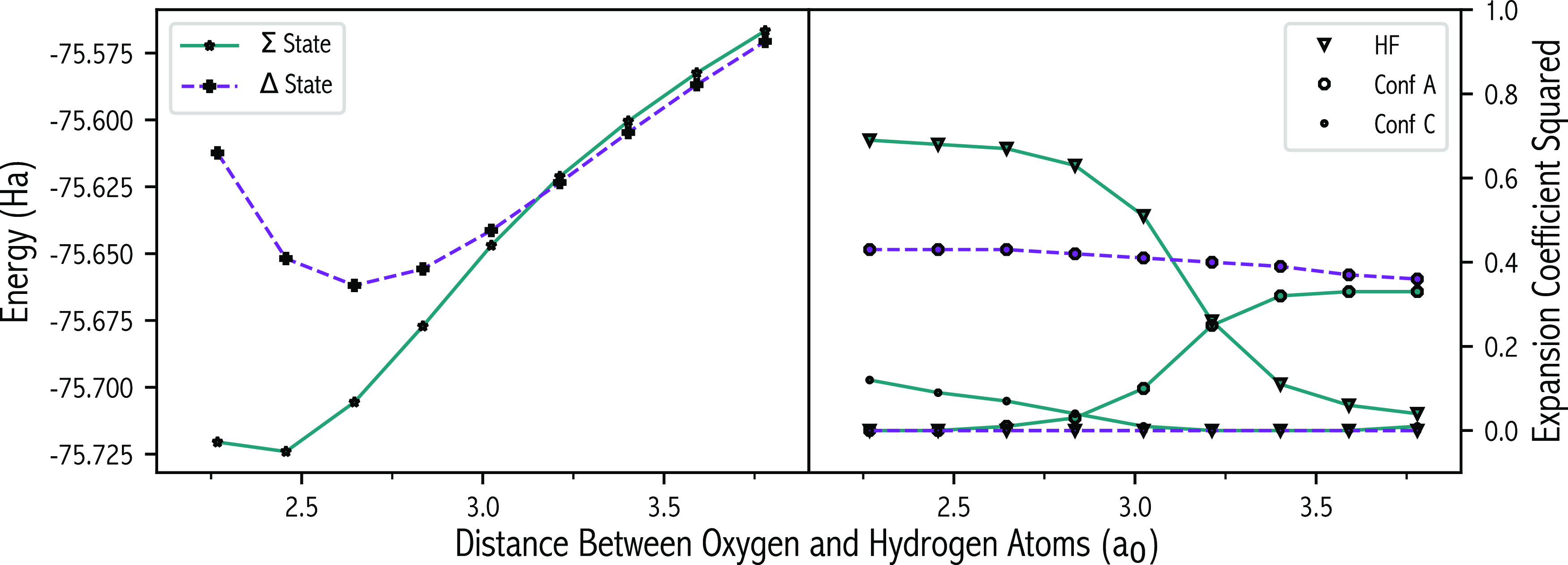
Energy of the ^1^Σ_g_^+^ and ^1^Δ_g_ states (left) and the expansion coefficient squared of the important configurations (right) as a function of the carbon–carbon distance, as computed using full configuration interaction.

C_2_ is a notorious problem in electronic-structure theory due to the unusually large MR character of the ^1^Σ_g_^+^ ground state at equilibrium, thus requiring sophisticated MR algorithms to qualitatively describe the electronic structure around regions that can normally be handled adequately by single-reference methods. FCI calculations^15^ have shown in 6-31G* that a crossing between the lowest-energy ^1^Σ_g_^+^ and ^1^Δ_g_ states, and an avoided crossing of the two lowest-energy ^1^Σ_g_^+^ states, exists. The crossing point (CP) of the ^1^Σ_g_^+^ and ^1^Δ_g_ states occurs between an internuclear separation of 3.02 and 3.21 a_0_ (see Figure 6). The FCI wave function contains 5.24 × 10^7^ SDs, approximately 3 times as many as that contained in the preceding FCI H_2_O wave function (^1^A_1_).

In accordance with previous studies,^15,102^ states were assigned based on the in-phase (^1^Σ_g_^+^) or out-of-phase (^1^Δ_g_) combination of the degenerate |2σ_g_^2^2σ_u_^2^1π_u(*y*)_^2^3σ_g_^2^⟩ (Conf A) and |2σ_g_^2^2σ_u_^2^1π_u(*x*)_^2^3σ_g_^2^⟩ (Conf B) configurations. The configurations are doubly excited relative to the HF configuration (|2σ_g_^2^2σ_u_^2^1π_u_^2^1π_u_^2^⟩) in which two electrons are excited from either the π_u(*x*)_ or the π_u(*y*)_ orbitals to the higher energy 3σ_g_ orbital. At each rCC the states were manually assigned by examining the coefficients of the aforementioned states.

In the FCI wave functions, the weighting of the degenerate Conf A and Conf B pair was identical (see Figure 6). However, for the approximate SCI methods, especially for small wave functions, this may not necessarily be true. In addition to the degenerate pair, the HF and |2σ_g_^2^1π_u(*x*)_^2^1π_u(*y*)_^2^3σ_g_^2^⟩ (Conf C) configurations have considerable weighting in the ^1^Σ_g_^+^ wave function.

The surface-averaged data for the approximated PESs discussed in this section are provided in Table S3, with a selected subset provided in Table 3. Despite one configurational space being optimized in a SA fashion for both roots, we average over the ^1^Σ_g_^+^ and ^1^Δ_g_ states individually when discussing the surface accuracy. Once again, to aid readability, the SA prefix is assumed when naming the methods.

**Table 3:** Nonparallelity Error (NPE) of the Carbon–Carbon Dissociation Potential Energy Surface for Various Selected Configuration Interaction Algorithms^a^

	X^1^Σ_g_^+^ state	B^1^Δ_g_ state	
algorithm	NPE	NPE	or
CASPT2(8,8)^b^^,^^102^	3.65	0.64	660
MRCISD^c^^,^^102^	0.17	0.32	270,388
SS-FCIQMC^d^^,^^25^	0.08		
SS-*i*-FCIQMC^d^^,^^25^	0.11		
SD-SA(2)-MCCI (10^–4^)	0.42/0.13	0.10/0.07	64,483
CSF-SA(2)-MCCI (10^–4^)	0.30/0.10	0.06/0.05	40,656
SD-SA(2)-XACI (σ = 6.28, 10^–^^4^)	0.22/0.47	0.75/0.38	14,589
SD-SA(2)-XACI (σ = 0.63, 10^–^^4^)	0.08/0.03	0.06/0.01	216,423
CSF-SA(2)-XACI (σ = 0.63, 10^–^^4^)	0.08/0.04	0.06/0.04	59,377
SD-SA(2)-XASCI (*n*_t_ = 5 × 10^4^, 10^–^^4^)	0.75/0.16	0.44/0.08	50,000
CSF-SA(2)-XASCI (*n*_t_ = 5 × 10^4^, 10^–^^4^)	0.24/0.05	0.22/0.04	50,000
SD-SA(2)-XHCI (ν = 0.31, 10^–^^4^)	0.61/0.23	0.66/0.18	46,311
CSF-SA(2)-XHCI (ν = 0.31, 10^–^^4^)	0.39/0.18	0.39/0.13	43,197

a The properties relating to the variational and non-variational energies are separated by /. All NPEs provided are in terms of kcal/mol, and  or  refers to the surface-averaged dimensionality of the variational wavefunction.

b The 2σ_g_, 2σ_u_, 1π_u(*x*)_, 1π_u(*y*)_, 1π_g(*x*)_, 1π_g(*y*)_, 3σ_g_, and 3σ_u_ orbitals were included in the active space.

c MRCISD using the aforementioned CASSCF(8,8) orbitals.

d The spherical cc-pVDZ basis set was used in this study. The crossing point of the X^1^Σ_g_^+^ and B^1^Δ_g_ states for all SCI algorithms included in this table was between 3.02 → 3.21 a_0_.

Previous SA(2)-CASPT2(8,8) calculations,^102^ which utilized the same valence active space as described earlier, have shown that this method struggles to describe the ^1^Σ_g_^+^ surface with the same level of accuracy as the ^1^Δ_g_ surface, with NPEs of 3.65 and 0.64 kcal/mol, respectively. In the same study, MR configuration interaction singles and doubles (MRCISD) using the CASSCF orbitals was shown to improve the surfaces, reducing NPEs to 0.17(^1^Σ_g_^+^) and 0.32(^1^Δ_g_) kcal/mol, resulting in two high-accuracy PESs. The low NPEs of the MRCISD surfaces suggest that the valence active space is a reasonable choice for this PES as poor performance would be expected if essentially CISD on a HF reference. The (*i*-)FCIQMC approach has also been shown to provide near-exact surfaces for the lowest energy state (state specific).^25^

**Figure 7:**
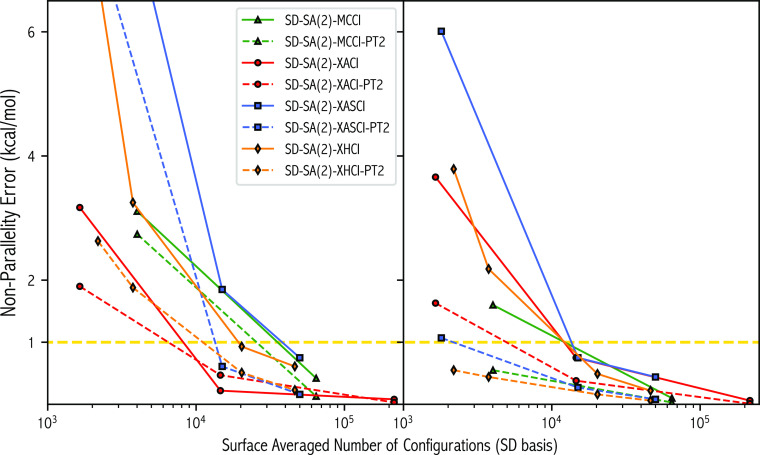
Nonparallelity error of the ^1^Σ_g_^+^ (left) and ^1^Δ_g_ (right) states as a function of the surface-averaged dimensionality of the converged variational wave function for various selected configuration algorithms and selection thresholds.

Despite being applied to two MR roots in a SA fashion, all SCI methods have the ability to once again generate very accurate PESs for both states, while remaining fairly compact. For this system, the largest  was 216,423 SDs [XACI(σ = 0.63, 10^–4^)], approximately 0.4% of the FCI space. The ζ_MR_ character of the SD-MCCI(10^–4^) wave function varied from 0.51 to 0.80 for the ^1^Σ_g_^+^ state and from 0.62 to 0.74 for the ^1^Δ_g_ state, showing clear MR character across the entirety of the surfaces for both states. The NPE as a function of  for the methods and various selection criteria is shown in Figure 7. Taking the SD-XACI(σ = 6.28, 10^–4^) as a starting point, NPEs of 0.22 and 0.75 kcal/mol were obtained for the ^1^Σ_g_^+^ and ^1^Δ_g_ states, respectively, for an  of 14,589 SDs. The  s of 7.06 (^1^Σ_g_^+^) and 6.24 (^1^Δ_g_) kcal/mol, 6.65 kcal/mol when averaged, are close to the σ value of 6.28 kcal/mol, and the CP was found to match that of FCI. For the ^1^Σ_g_^+^ state, SD-MCCI(10^–4^), SD-XASCI(*n*_t_ = 5 × 10^4^, 10^–4^), and SD-XHCI(ν = 0.31, 10^–4^) have larger NPEs despite significantly larger . However, the opposite is observed for the ^1^Δ_g_ state as SD-XACI(σ = 6.28, 10^–4^) struggles to deal with this state at the same level of accuracy/size as the ^1^Σ_g_^+^ state. Lowering the XACI selection threshold, SD-XACI(σ = 0.63, 10^–4^), leads to a drastic increase in the dimensionality of the XACI wave functions,  of 216,423 SDs, and results in near-exact surfaces (NPEs less than 0.1 kcal/mol). The SD-XACI(σ = 0.63, 10^–4^) surface has the largest dimensionality, by a considerable amount, for the range of selection thresholds and methods tested. These NPEs are lower than MRCISD, which also benefits from the use of CASSCF orbitals, and very similar to (*i*)-FCIQMC. With the exception of SD-XASCI(*n*_t_ = 1.8 × 10^3^, 10^–4^) and SD-XHCI(ν = 6.28, 10^–4^), which are among some of the smallest wave functions encountered, all SCI methods and selection thresholds are able to accurately reproduce the FCI CP. Interestingly, XACI(σ = 6.28, 10^–4^), which has a smaller  when compared to the a for mentioned XASCI and XHCI surfaces can reproduce the FCI CP. Once again, inclusion of the PT2 correction has a considerable effect on NPEs, which is also true for XACI when ran in a SA fashion. The  was computed for the SD-XACI(σ = 6.28, 10^–4^) wave functions and was found to be close to 0 across the entirety of the PES, therefore spin-augmentation was not required for the range of rCC separations considered.

In order to investigate the compactness of the CSF basis for this system, the surfaces for the tightest selection thresholds were recomputed using CSFs (see Figure S3). Once again, for XACI a drastic decrease in the number of configurations is observed,  of 216,423 SDs compared to a  of 59,377 CSFs ( of 3.6:1) bringing them inline with the other approaches. For MCCI, we also observe a decrease in , going from 64,483 SDs to 40,656 CSFs ( of 1.6:1), albeit not as drastic as that observed for XACI. For XHCI, the dimensionality in both bases was very similar,  and  of 46,311 SDs and 43,197 CSFs, respectively. For all methods and selection criteria, the NPEs computed using CSFs were lower than their SD counterpart with the exception of XACI(σ = 0.63, 10^–4^) which gave identical NPEs.

### 5.3 CH_4_ Dissociation

The final potential energy surface of interest is the single C–H bond breaking pathway in CH_4_. While we expect this surface to be less MR than the previous PESs investigated, the main goal of this section is to briefly explore the applicability of the SCI approaches to FCI spaces which approach 10^9^ SDs. The FCI space of the A′_g_ wave function in cc-pVDZ contains over 8.37 × 10^8^ SDs, which is almost 16 times larger than the FCI space of C_2_.

**Figure 8:**
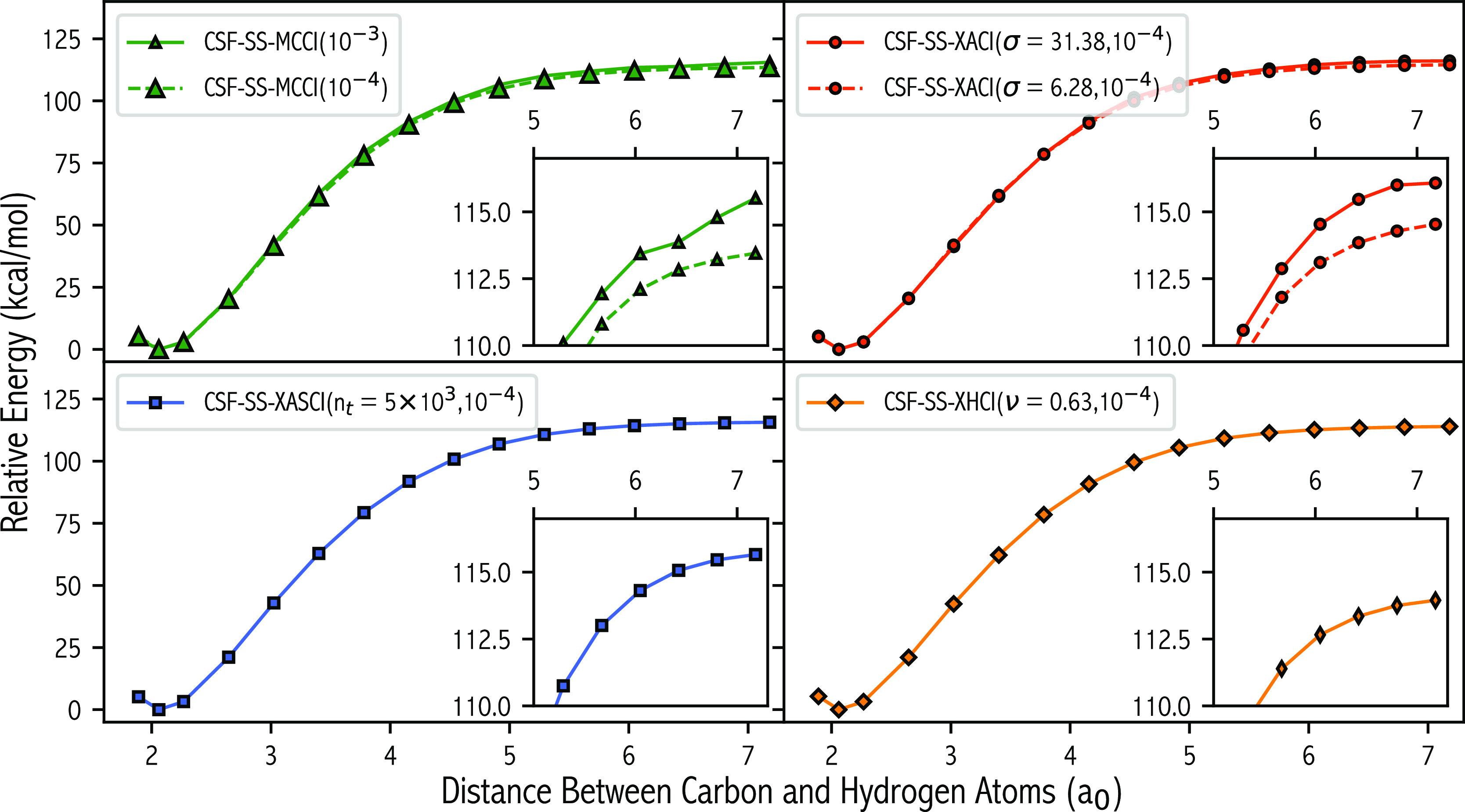
Relative energy of the A′ state as a function of the distance of a single carbon–hydrogen distance for various selected configuration interaction algorithms and selection thresholds. All other bonds remained frozen across the surface.

In this section, we compare the dissociation energy (*D*_e_) for each of the methods (see Table 4), which is computed as the energy difference between an rCH of 1.8 and 7.18 a_0_. At FCI, *D*_e_ was predicted to be 113.74 kcal/mol. For CSF-XACI(σ = 31.38, 10^–4^),  is extremely small at 375 CSFs, which is approximately 4.5 × 10^–5^% of the FCI space. Despite such a small configurational space the PES is very smooth (see Figure 8), *D*_e_ was predicted to be 116.08 kcal/mol which is approximately 2 kcal/mol larger than FCI. Decreasing σ to 6.28 kcal/mol, increases  by an order of magnitude (3115 CSFs) and reduces *D*_e_ to 114.54 kcal/mol, which is within chemical accuracy. For SD-XACI(σ = 6.28, 10^–4^),  increased to 11,424 SDs ( of 3.7:1) and *D*_e_ lowered by around 0.5 kcal/mol (*D*_e_ = 114.03 kcal/mol) giving excellent agreement to FCI. The ζ_MR_ of the SD-XACI(σ = 6.28, 10^–4^) wave function at an rCC of 1.8 and 7.18 a_0_ was found to be 0.124 and 0.615, respectively. This shows that despite having a much larger FCI space, the MR character of the dissociated species is less than that of H_2_O, owed to the fact that only one bond is being broken in this PES. For CSF-MCCI(10^–3^), small discontinuities were observed for large separations, which was rectified when transitioning to a *c*_min_ of 10^–4^. We attribute this to the stochastic element in MCCI and the larger FCI space for this system; therefore, it is entirely possible that important configurations may be missed. The *D*_e_ predicted at the two different *c*_min_ thresholds was 115.1 (10^–3^) and 113.45 (10^–4^) kcal/mol, with  increasing from 2547 to 39,305 CSFs. The surface predicted by XASCI(*n*_t_ = 5 × 10^3^, 10^–4^) is also very smooth, with a *D*_e_ of 115.66 kcal/mol, which is 1 kcal/mol further away from FCI when compared to the smaller  CSF-XACI(σ = 6.28, 10^–4^) surface. The *D*_e_ predicted by CSF-XHCI(ν = 0.63, 10^–4^), 113.95 kcal/mol, gave the best agreement to FCI, deviating by only 0.21 kcal/mol, despite a relatively small  of 7642 CSFs.

**Table 4:** Predicted Dissociation Energy (*D*_e_) for a Single Hydrogen–Carbon Dissociation in CH_4_^a^

algorithm	*D*_e_	or
FCI	113.74	8.37 × 10^8^
CSF-SS-MCCI (10^–3^)	115.1	2547
CSF-SS-MCCI (10^–4^)	113.45	39,305
CSF-SS-XACI (σ = 31.38, 10^–^^4^)	116.08	375
CSF-SS-XACI (σ = 6.28, 10^–^^4^)	114.54	3115
SD-SS-XACI (σ = 6.28, 10^–^^4^)	114.03	11,424
CSF-SS-XASCI (*n*_t_ = 5 × 10^3^, 10^–^^4^)	115.66	5000
CSF-SS-XHCI (ν = 0.63, 10^–^^4^)	113.95	7642

a All energies provided are in terms of kcal/mol and  or  refers to the surface-averaged dimensionality of the variational wavefunction.

## 6 Conclusions

In this work, we have outlined a recently developed selected configuration interaction (SCI) code which is built using the existing Monte-Carlo configuration interaction (MCCI) code.^52,66^ This SCI code allows for fair comparison of wave functions generated by different selection criteria, and the modular framework facilitates the implementation of new selection protocols. All SCI algorithms follow the same series of steps and only differ by the specific selection protocol, which imposes the constraints which are used to build the SCI wave function. As a starting point, we have implemented variations of the adaptive configuration interaction (ACI),^34,53^ adaptive sampling configuration interaction (ASCI),^33^ and heat-bath configuration interaction (HCI)^32^ algorithms and performed a thorough comparison. Additionally, to the best of our knowledge, we also provide the first implementation of such algorithms in the basis of configuration state functions (CSFs), allowing one to take advantage of the compactness of such a representation when compared to the basis of Slater determinants (SDs). This is particularly true for the ACI algorithm in which wave functions were found to be vastly reduced, by up to 73%, when compared to the equivalent calculation in the SD basis. In the SD basis, we have also implemented a recently proposed spin-adaption algorithm^89^ to ensure convergence to spin-pure states. Additionally, the algorithms can also be run in a state-specific or state-averaged regime depending on the user’s needs.

We have compared our implementation of these SCI algorithms, denoted by the X prefix, in their ability to predict complicated bond-breaking potential energy surfaces (PESs), compared to full configuration interaction (FCI) and conventional post-Hartree–Fock methods, when applicable. First, predicted PESs for the double-hydrogen dissociation in H_2_O for various spin manifolds (singlet, triplet, and quintet) were compared. All methods were found to give extremely small nonparallelity errors (NPEs) for compact wave functions. In particular, the error-controlled XACI method was found to be extremely compact giving an NPE of less than 1 kcal/mol for a surface-averaged wave function dimensionality of 950 CSFs (approximately 0.02% of the FCI CSF space). To put this into context, the FCI space for this system contained over 19 million SDs. For state-specific calculations, the XACI method, in terms of surface accuracy, was also found to be essentially invariant to second-order perturbative corrections, which was found to be more important for the other SCI algorithms. The use of restricted Hartree–Fock and approximate natural orbitals were compared for the SCI methods, with the latter orbital basis providing a sizable reduction in the dimensionality and energy of the wave function. Due to the error-control selection procedure, the XACI method only benefits from the reduced dimensionality.

The crossing of the ^1^Σ_g_^+^ and ^1^Δ_g_ states in C_2_ was then explored in a state-averaged fashion, with all SCI methods outperforming CASPT2(8,8) surfaces and rivalling those predicted by MRCISD^102^ (valence active space), for much reduced wave functions. In particular, the NPEs of the variational energy of both surfaces predicted by the XACI algorithm in the CSF basis were found to be less than 0.1 kcal/mol despite only containing on average 59,377 CSFs. This bettered MRCISD and rivalled state-specific FCI quantum Monte-Carlo.^25^

Finally, the dissociation energy (*D*_e_) of breaking a single carbon–hydrogen bond in CH_4_ was explored (*D*_e_ = 113.74 kcal/mol). This problem was chosen as it has a relatively large FCI space, 837 million SDs, allowing us to explore the use of these SCI algorithms for this relatively large configurational space. Despite the large FCI space, the SCI methods are once again able to provide very accurate yet compact wave functions. Notably, the XACI method was found to give a *D*_e_ within chemical accuracy despite containing on average 3115 CSFs (*D*_e_ of 114.54 kcal/mol). The XHCI algorithm was found to give the best agreement to FCI (113.95 kcal/mol) despite using a relatively large selection threshold giving fairly modest wave functions. Interestingly, despite such a large FCI space, the stochastic MCCI approach also shows good accuracy (*D*_e_ = 113.45 kcal/mol), with the benefit of not having to generate the entire singles and doubles space relative to a reference, which is needed in all other approaches.

By considering purely the selection protocols on the systems and basis sets in this work, we find that all the methods can give accurate results using a small fraction of the FCI space. For PESs, the XACI approach of approximately controlling the energy error usually gave the best ratio of accuracy to wave function size. However, we emphasize that the original implementations of these methods exploit various ways of making the SCI calculations more efficient and we focused not on timings in this work but on a fair comparison of the selection criteria.

Recently, the efficient calculation of two-electron reduced density matrices and analytic gradients was proposed and implemented by refs (103) and (104), which can be extended to any SCI wave function. Our current research interest stems in using these compact, accurate wave functions as the electronic component in quantum dynamics, eliminating the need to use active-space dependent methods.

## Supporting Information Available

All surface averaged properties of interest for the range of selection thresholds and theories investigated. The SD-to-CSF ratio for selected methods. The MR character and total-spin squared for SD-SS-XACI (σ = 6.28, 10^–4^) as a function of rOH (PDF)
