# Supplementary material for: Modular Approach to Selected Configuration Interaction in an Arbitrary Spin Basis: Implementation and Comparison of Approaches
Source: J Chem Theory Comput. 2023 Dec 7;19(24):9161–76. doi: 10.1021/acs.jctc.3c00897 (PMC10753805; doi:10.1021/acs.jctc.3c00897)
Supplement: Supplementary file 1 — ct3c00897_si_001.pdf [file ct3c00897_si_001.pdf]

A Modular Approach to Selected Configuration Interaction in an Arbitrary Spin Basis: Implementation and Comparison of Approaches

Andrew W. Prentice,* Jeremy P. Coe, and Martin J. Paterson*

Institute of Chemical Sciences, School of Engineering and Physical Sciences, Heriot-Watt University, EH14 4AS, UK

E-mail: a.prentice@hw.ac.uk; m.j.paterson@hw.ac.uk

H_2O

Whilst performing well around equilibrium, CC theories are unable to describe the double bond breaking $^1A_1$ PES to high accuracy with NPEs of 13.07 and 59.45 kcal/mol for coupled cluster singles and doubles (CCSD) and the perturbative triples extension [CCSD(T)], respectively. For CASSCF(8,7), large NPEs were observed which drastically decreased when including dynamic correlation (CASPT2), giving NPEs of 3.07, 2.24 and 0.96 kcal/mol for the $^1A_1$, $^3A_1$ and $^5A_1$ states, respectively.

Table S1: Various properties of the predicted double-hydrogen dissociation PES in H_2O in various spin manifolds. When applicable, the properties relating to the variational and non-variational energies are separated by /. The NPE,σ and $\overline{|\Delta FCI|}$ values provided are in terms of kcal/mol and $\overline{SD}$ or $\overline{CSF}$ refers to the surface-averaged dimensionality of the variational wavefunction.

	$^1A_1$ State				$^3A_1$ State				$^5A_1$ State			
Algorithm	NPE	$\overline{\lvert\Delta FCI\rvert}$	σ_{SD}	$\overline{SDs}$ or $\overline{CSFs}$	NPE	$\overline{\lvert\Delta FCI\rvert}$	σ_{SD}	$\overline{SDs}$ or $\overline{CSFs}$	NPE	$\overline{\lvert\Delta FCI\rvert}$	σ_{SD}	$\overline{SDs}$ or $\overline{CSFs}$
FCI	0.00	0.00	0.00	1.9604×10^7	0.00	0.00	0.00	1.4888×10^7	0.00	0.00	0.00	6.377×10^6
CCSD	13.07	8.30	4.34	837	–	–	–	–	–	–	–	–
CCSD(T)	59.45	15.66	21.22	837	–	–	–	–	–	–	–	–
CASSCF[a]/CASPT2[b]	25.70/3.06	80.92/6.46	9.47/1.22	149	28.96/2.24	81.84/5.88	10.90/0.77	141	13.55/0.96	75.14/5.74	4.74/0.28	27
CSF-SS-MCCI(10^{-3})	3.48/1.08	7.58/1.77	0.92/0.31	1287	2.91/1.56	8.63/1.21	0.81/0.79	1853	5.54/0.36	4.97/0.57	1.65/0.11	1315
CSF-SS-MCCI(10^{-4})	0.65/0.24	0.78/0.22	0.23/0.08	16526	0.69/0.16	0.98/0.12	0.26/0.09	19330	0.68/0.02	0.56/0.04	0.25/0.00	12401
SD-SS-MCCI(10^{-4})	0.91/0.64	1.36/0.13	0.34/0.19	25657	0.85/0.03	1.07/0.04	0.33/0.01	21666	0.74/0.04	0.55/0.02	0.27/0.01	12553
CSF-SS-XACI($\sigma = 31.38,10^{-4}$)	5.99/6.29	41.27/10.07	1.82/1.87	227	11.95/10.66	36.99/6.29	2.83/2.57	349	1.84/1.60	34.54/3.72	0.50/0.54	149
CSF-SS-XACI($\sigma = 6.28,10^{-4}$)	0.77/0.75	8.67/2.40	0.22/0.22	950	3.17/2.64	7.75/1.57	1.07/1.07	2071	0.97/1.02	7.71/1.46	0.31/0.33	986
SD-SS-XACI($\sigma = 6.28,10^{-4}$)	0.92/0.73	6.68/0.44	0.29/0.29	3152	0.39/0.39	6.70/0.42	0.11/0.11	2348	0.13/0.14	6.63/0.36	0.03/0.03	1044
SD-SS-XACI($\sigma = 6.28,10^{-4}$)[c]	0.40/0.54	6.12/0.33	0.11/0.22	5314	–	–	–	–	–	–	–	–
CSF-SS-XACI($\sigma = 0.63,10^{-4}$)	0.14/0.12	0.94/0.30	0.04/0.04	14375	0.24/0.21	0.80/0.18	0.08/0.07	27713	0.11/0.10	0.73/0.10	0.04/0.04	11678
SD-SS-XACI($\sigma = 0.63,10^{-4}$)	0.05/0.03	0.67/0.02	0.01/0.01	52093	0.03/0.01	0.67/0.03	0.01/0.00	36293	0.02/0.01	0.66/0.03	0.01/0.00	12278
CSF-SS-XASCI(adaptive-n_t,10^{-4})[d]	1.59/0.78	8.24/2.23	0.47/0.21	950	–	–	–	–	–	–	–	–
CSF-SS-XASCI($n_t = 10^3,10^{-4}$)	6.05/1.69	7.97/2.19	2.10/0.56	1000	10.17/3.23	12.50/2.13	3.28/1.03	1000	12.52/1.35	6.95/0.95	4.50/0.49	1000
CSF-SS-XASCI($n_t = 10^4,10^{-4}$)	1.99/0.61	1.40/0.42	0.69/0.22	10000	2.22/0.29	1.80/0.22	0.75/0.18	10000	1.72/0.16	0.78/0.07	0.59/0.05	10000
CSF-SS-XASCI($n_t = 5\times10^4,10^{-4}$)	0.44/0.17	0.25/0.09	0.15/0.06	50000	0.64/0.09	0.41/0.08	0.23/0.03	50000	0.35/0.04	0.15/0.02	0.13/0.01	50000
SD-SS-XASCI($n_t = 5\times10^4,10^{-4}$)	1.02/0.03	0.69/0.02	0.36/0.02	50000	0.80/0.03	0.47/0.02	0.29/0.01	50000	0.35/0.02	0.14/0.01	0.13/0.01	50000
CSF-SS-XHCI($\nu = 0.63,10^{-4}$)	1.86/0.62	2.05/0.57	0.67/0.21	9048	2.28/0.33	2.30/0.25	0.78/0.25	11634	1.53/0.09	1.44/0.09	0.55/0.02	7147
CSF-SS-XHCI($\nu = 0.31,10^{-4}$)	0.90/0.30	0.89/0.25	0.31/0.10	21559	1.23/0.19	1.09/0.11	0.44/0.11	27321	0.89/0.02	0.69/0.03	0.32/0.01	15765
CSF-SS-XHCI($\nu = 0.06,10^{-4}$)	0.18/0.07	0.13/0.04	0.06/0.02	87223	0.20/0.01	0.16/0.01	0.07/0.01	136145	0.15/0.00	0.10/0.00	0.05/0.00	76051
SD-SS-XHCI($\nu = 0.06,10^{-4}$)	0.34/0.02	0.30/0.01	0.11/0.01	135777	0.29/0.01	0.25/0.01	0.10/0.00	110454	0.20/0.01	0.13/0.01	0.07/0.00	59310

[a] The $2a_1$, $3a_1$, $4a_1$, $1b_1$, $1b_2$, $2b_2$ and $3b_2$ orbitals were included in the active space. [b] The same active space as CASSCF(8,7) was used with a level shift of 0.3.[1,c] The spin-corrected procedure was implemented across all points. [d] The n_t parameter was varied to match the number of configurations in the converged CSF-SS-XACI(6.28,10^{-4}) wavefunction.

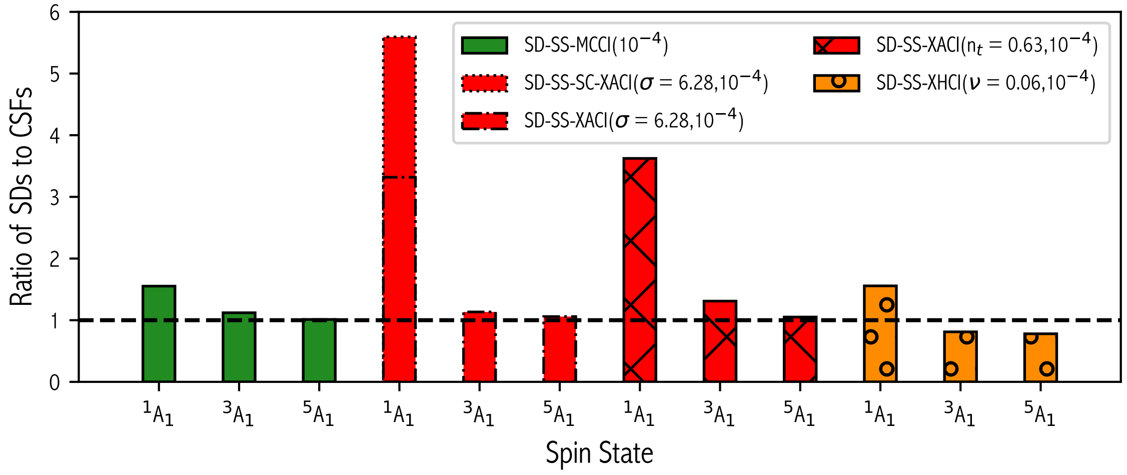

Figure S1: The ratio of the average number of Slater determinants to configuration state functions across the H_2O bond-breaking surface for various electronic-structure theories. The horizontal line highlights a ratio of one, where the number of SDs and CSFs are equal.

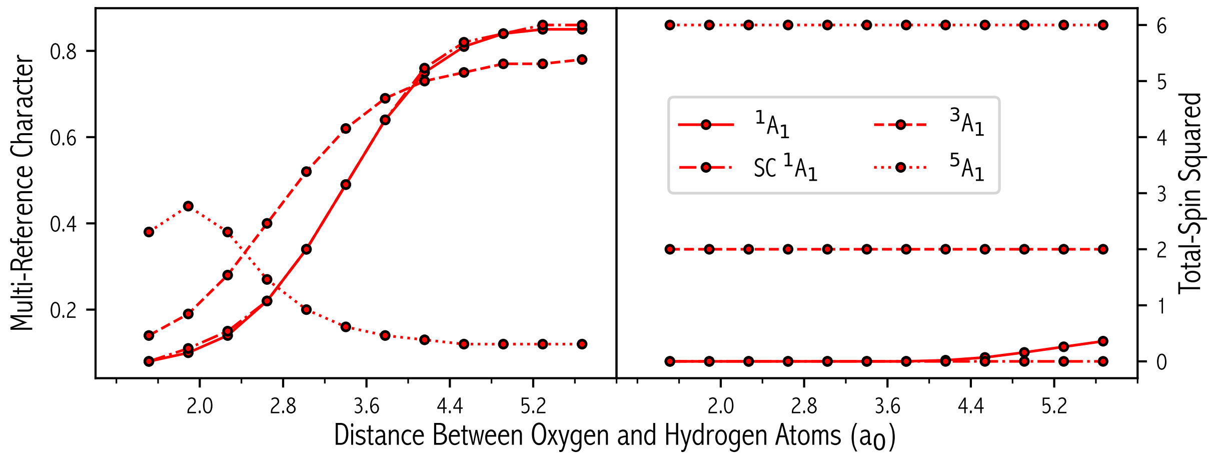

Figure S2: The multi-reference character (left) and the total-spin squared (right) of the $^1A_1$, $^3A_1$ and $^5A_1$ states as a function of both oxygen-hydrogen distances predicted using SD-SS-XACI($\sigma = 6.28, 10^{-4}$). The spin-corrected (SC) SD-SS-XACI($\sigma = 6.28, 10^{-4}$) values are also presented.

Table S2: The energy difference, with respect to full configuration interaction (ΔFCI), and the dimensionality of the variational wavefunction (CSF basis) predicted in the canonical restricted Hartree-Fock or approximate natural orbital basis for various selected configuration interaction algorithms. All energies are given in terms of kcal/mol. Both oxygen-hydrogen distances were set to 4.54 a_0 and only the $^1A_1$ state is considered.

	RHF Orbitals		MCCI(10^{-3}) NOs	
Algorithm	ΔFCI	CSFs	ΔFCI	CSFs
CSF-MCCI(10^{-4})	0.65	13182	0.43	7168
CSF-XACI($\sigma = 6.28$,10^{-4})	8.72	910	8.37	414
CSF-XACI($\sigma = 0.63$,10^{-4})	0.91	8553	0.88	3187
CSF-XASCI($n_t = 10^3$,10^{-4})	7.61	1000	2.52	1000
CSF-XASCI($n_t = 10^4$,10^{-4})	0.81	10000	0.18	10000
CSF-XASCI($n_t = 5 \times 10^4$,10^{-4})	0.12	50000	0.03	50000
CSF-XHCI($\nu = 0.63$,10^{-4})	1.52	8472	0.60	6246
CSF-XHCI($\nu = 0.06$,10^{-4})	0.64	72817	0.04	40680

C_2

Table S3: Various properties of the predicted carbon-carbon dissociation PESs in C_2. When applicable, the properties relating to the variational and non-variational energies are separated by /. The NPE, σ and $\overline{|\Delta \text{FCI}|}$ values provided are in terms of kcal/mol and $\overline{\text{SD}}$ or $\overline{\text{CSF}}$ refers to the surface-averaged dimensionality of the variational wavefunction.

	$X^1\Sigma_g^+$ State			$B^1\Delta_g$ State				
Algorithm	NPE	$\overline{\lvert\Delta\text{FCI}\rvert}$	σ_{SD}	NPE	$\overline{\lvert\Delta\text{FCI}\rvert}$	σ_{SD}	$\overline{\text{SDs}}$ or $\overline{\text{CSFs}}$	Crossing Point
FCI	–	–	–	–	–	–	5.24×10^7	3.02→3.21
SA-CASSCF(8,8)[a]/CASPT2(8,8)[2]	5.42/3.65	74.24/5.67	2.24/1.45	4.65/0.64	78.67/7.53	1.52/0.20	660	3.21→3.40/3.02→3.21
MRCISD[b, 2]	0.17	1.59	0.05	0.32	1.55	0.11	270388	3.02→3.21
SS-FCIQMC[c, 3]	0.08	0.05	0.02	–	–	–	–	–
SS-*i*-FCIQMC[c, 3]	0.11	0.06	0.04	–	–	–	–	–
SD-SA(2)-MCCI(10^{-3})	3.11/2.74	16.10/1.76	1.06/0.89	1.60/3.42	13.78/1.36	0.55/0.99	4011	3.02→3.21/3.02→3.21
SD-SA(2)-MCCI(10^{-4})	0.42/0.13	2.48/0.12	0.13/0.05	0.10/0.07	2.15/0.07	0.03/0.02	64483	3.02→3.21/3.02→3.21
CSF-SA(2)-MCCI(10^{-4})	0.30/0.10	1.50/0.36	0.09/0.04	0.06/0.05	1.30/0.32	0.02/0.02	40656	3.02→3.21/3.02→3.21
SD-SA(2)-XACI($\sigma = 31.38, 10^{-4}$)	3.17/1.90	34.27/1.74	0.87/0.64	3.66/1.63	32.11/1.75	1.36/0.57	1645	3.02→3.21/3.02→3.21
SD-SA(2)-XACI($\sigma = 6.28, 10^{-4}$)	0.22/0.47	7.06/0.44	0.06/0.15	0.75/0.38	6.24/0.49	0.27/0.13	14589	3.02→3.21/3.02→3.21
SD-SA(2)-XACI($\sigma = 0.63, 10^{-4}$)	0.08/0.03	0.78/0.04	0.02/0.01	0.06/0.01	0.91/0.28	0.02/0.00	216423	3.02→3.21/3.02→3.21
CSF-SA(2)-XACI($\sigma = 0.63, 10^{-4}$)	0.08/0.04	1.04/0.31	0.03/0.02	0.06/0.04	0.91/0.28	0.02/0.01	59377	3.02→3.21/3.02→3.21
SD-SA(2)-XASCI($n_t = 1.8 \times 10^3, 10^{-4}$)	11.05/8.14	27.08/3.16	3.86/2.31	6.01/1.07	21.68/1.22	2.15/0.35	1800	2.83→3.02/2.83→3.02
SD-SA(2)-XASCI($n_t = 1.5 \times 10^4, 10^{-4}$)	1.85/0.61	7.10/0.52	0.64/0.21	0.75/0.27	5.97/0.26	0.27/0.09	15000	3.02→3.21/3.02→3.21
SD-SA(2)-XASCI($n_t = 5 \times 10^4, 10^{-4}$)	0.75/0.16	3.02/0.16	0.25/0.05	0.44/0.08	2.61/0.09	0.16/0.03	50000	3.02→3.21/3.02→3.21
CSF-SA(2)-XASCI($n_t = 5 \times 10^4, 10^{-4}$)	0.24/0.05	1.21/0.33	0.08/0.02	0.22/0.04	1.07/0.30	0.08/0.02	50000	3.02→3.21/3.02→3.21
SD-SA(2)-XHCI($\nu = 6.28, 10^{-4}$)	6.88/2.63	31.54/1.80	1.99/0.84	10.48/1.70	26.24/1.68	3.79/0.55	2181	2.83→3.02/3.02→3.21
SD-SA(2)-XHCI($\nu = 3.14, 10^{-4}$)	3.25/1.88	20.40/1.26	0.92/0.58	6.51/1.28	16.89/1.07	2.18/0.44	3752	3.02→3.21/3.02→3.21
SD-SA(2)-XHCI($\nu = 0.63, 10^{-4}$)	0.93/0.51	6.92/0.34	0.30/0.17	1.44/0.46	6.60/0.30	0.49/0.16	20314	3.02→3.21/3.02→3.21
SD-SA(2)-XHCI($\nu = 0.31, 10^{-4}$)	0.61/0.23	4.00/0.16	0.20/0.08	0.66/0.18	3.74/0.14	0.22/0.06	46311	3.02→3.21/3.02→3.21
CSF-SA(2)-XHCI($\nu = 0.31, 10^{-4}$)	0.39/0.18	1.99/0.46	0.14/0.07	0.39/0.13	1.75/0.42	0.12/0.04	43197	3.02→3.21/3.02→3.21

[a] The $2\sigma_g$, $2\sigma_u$, $1\pi_{u(x)}$, $1\pi_{u(y)}$, $1\pi_{g(x)}$, $1\pi_{g(y)}$, $3\sigma_g$ and $3\sigma_u$ orbitals were included in the active space. [b] MRCISD using the CASSCF(8,8) orbitals. [c] The spherical cc-pVDZ basis set was used in this study.

References

(1) Roos, B. O.; Andersson, K. Multiconfigurational perturbation theory with level shift — the Cr_2 potential revisited. *Chemical Physics Letters* **1995**, *245*, 215–223.

(2) Sherrill, C. D.; Piecuch, P. The $X\ ^1\Sigma_g^+$, $B\ ^1\Delta_g$, and $B'\ ^1\Sigma_g^+$ states of C_2: A comparison of renormalized coupled-cluster and multireference methods with full configuration interaction benchmarks. *The Journal of Chemical Physics* **2005**, *122*, 124104.

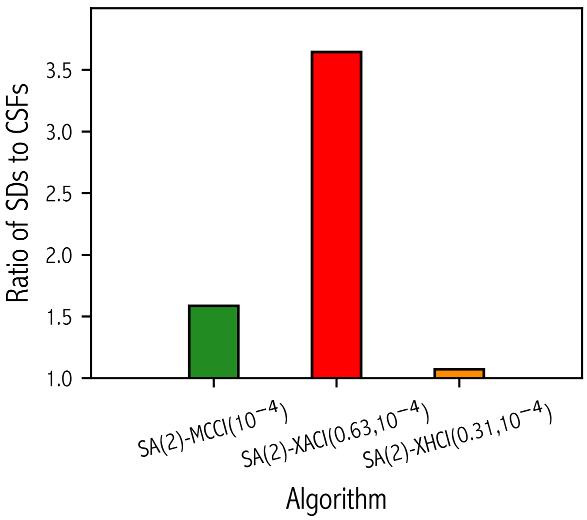

Figure S3: The ratio of the average number of Slater determinants to configuration state functions across the C_2 bond-breaking surface for various electronic-structure theories.

(3) Booth, G. H.; Cleland, D.; Thom, A. J. W.; Alavi, A. Breaking the carbon dimer: The challenges of multiple bond dissociation with full configuration interaction quantum Monte Carlo methods. *The Journal of Chemical Physics* **2011**, *135*, 084104.
